# Non-CG DNA methylation is a biomarker for assessing endodermal differentiation capacity in pluripotent stem cells

**DOI:** 10.1038/ncomms10458

**Published:** 2016-01-29

**Authors:** Lee M. Butcher, Mitsuteru Ito, Minodora Brimpari, Tiffany J. Morris, Filipa A. C. Soares, Lars Ährlund-Richter, Nessa Carey, Ludovic Vallier, Anne C. Ferguson-Smith, Stephan Beck

**Affiliations:** 1 UCL Cancer Institute, University College London, 72 Huntley Street, London WC1E 6BT, UK; 2 Department of Surgery and Cancer, Imperial College London, London W12 0NN, UK; 3 Department of Genetics, University of Cambridge, Cambridge CB2 3EH, UK; 4 Anne McLaren Laboratory, Department of Surgery, Wellcome Trust and Medical Research Council Stem Cell Institute, University of Cambridge, Cambridge CB2 0SZ, UK; 5 Cambridge Epigenetix, Jonas Webb Building, Babraham Campus, Cambridge CB22 3AT, UK; 6 Wellcome Trust Sanger Institute, Hinxton CB10 1SA, UK; 7 Division of Paediatric Oncology, Department of Women's and Children's Health, Karolinska Institutet,171 76 Stockholm, Sweden; 8 PraxisUnico, The Jeffreys Building, St John's Innovation Park, Cambridge CB4 0DE, UK

## Abstract

Non-CG methylation is an unexplored epigenetic hallmark of pluripotent stem cells. Here we report that a reduction in non-CG methylation is associated with impaired differentiation capacity into endodermal lineages. Genome-wide analysis of 2,670 non-CG sites in a discovery cohort of 25 phenotyped human induced pluripotent stem cell (hiPSC) lines revealed unidirectional loss (Δ*β*=13%, *P*<7.4 × 10^−4^) of non-CG methylation that correctly identifies endodermal differentiation capacity in 23 out of 25 (92%) hiPSC lines. Translation into a simplified assay of only nine non-CG sites maintains predictive power in the discovery cohort (Δ*β*=23%, *P*<9.1 × 10^−6^) and correctly identifies endodermal differentiation capacity in nine out of ten pluripotent stem cell lines in an independent replication cohort consisting of hiPSCs reprogrammed from different cell types and different delivery systems, as well as human embryonic stem cell (hESC) lines. This finding infers non-CG methylation at these sites as a biomarker when assessing endodermal differentiation capacity as a readout.

## Abstract (web summary)

The methylation of non-CpG residues is a poorly understood marker of pluripotent cells, gradually lost as cells differentiate. Here the authors show non-CG methylation can be used as a marker of differentiation potential.

## Graphical abstract

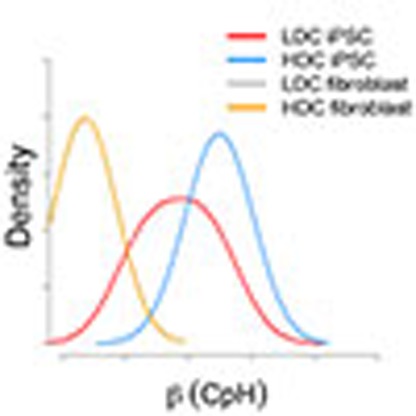

Non-CG DNA methylation is an epigenetic hallmark of mammalian pluripotent stem cells^1^, which is gradually lost on cell fate specification^2^,^3^. The occurrence of non-CG methylation is predominantly catalysed by *DNMT3A* and *DNMT3B* in cooperation with *DNMT3L*^2^,^3^,^4^ and was first described in embryonic stem cells; however, its prevalence and genomic localization has only been established recently^5^,^6^. Unlike patterns of CG methylation where the majority of cytosines are methylated^6^,^7^,^8^ and have been both extensively studied^9^,^10^,^11^,^12^ and reviewed^13^,^14^,^15^, cytosines at non-CG dinucleotides in pluripotent stem cell populations are only partially methylated and less well studied^4^,^6^,^8^,^16^. We hypothesized that quantitative variation in non-CG methylation may reflect pluripotent stem cell-specific phenotypes and thus be suitable as a biomarker capable of predicting differentiation capacity, complementing existing methods such as PluriTest^17^ (which uses gene expression data) and Scorecard^9^ (which uses CpG methylation and gene expression data). Identification of a biomarker solely based on few non-CG sites would thus constitute an alternative. The importance of discovering robust and easy-to-assay biomarkers is that time-consuming and costly cell-based phenotyping measures can be reduced, while simultaneously offering improvements in large-scale assessment of human induced pluripotent stem cell (hiPSC) lines for clinical utility and therapeutic applications.

Using a two-stage design we report here that pluripotent stem cells with an impaired differentiation capacity for endodermal lineages exhibit reduced DNA methylation at non-CG dinucleotides targeted by the Illumina 450K array. This finding infers non-CG methylation can be used as a biomarker for assessing endodermal differentiation capacity.

## Results

### Differentiation capacity in pluripotent stem cell lines

We used a two-stage design consisting of a discovery cohort of 25 hiPSC lines, and a replication cohort of 7 hiPSC lines from the HipSci study (http://www.hipsci.org) and 3 human embryonic stem cell (hESC) lines (see Supplementary Table 1). In the discovery cohort, we analysed hiPSC lines derived from a range of differentiated somatic cells (dermal fibroblasts (*N*=20), foreskin fibroblasts (*N*=1), endothelial precursor cells (*N*=2) and endothelial progenitor cells (*N*=2)) obtained from seven unrelated individuals and two monozygotic twins. These hiPSC lines were derived using *OCT4*, *SOX2*, *KLF4* and *c-MYC* (OSKM) reprogramming factors delivered using two virus-based vectors (retrovirus (*N*=23) and Sendai virus (*N*=2)). In the replication cohort, we extended the source of pluripotent stem cells to include hESCs as well as diversifying the range of donor cell types and OSKM delivery methods from which hiPSC lines were derived; donor cells included erythroblasts (*N*=4) and dermal fibroblasts (*N*=*3*) obtained from five unrelated individuals, and OSKM delivery systems included a DNA-based vector (episomal (*N*=3)) in addition to a virus-based vector (Sendai virus (*N*=4)); confirmation of pluripotency, differentiation protocol and differentiation capacity scoring are detailed in Methods. For the hiPSCs used in the discovery cohort and hESC lines in the replication cohort, we obtained multiple DNA extracts from independent cells of the same plate (median *N*=6), to control for cell line variation (Supplementary Fig. 1); for the hiPSC lines in the replication cohort, we obtained only single DNA extracts per plate. All hiPSC lines in the discovery and replication cohorts fulfilled criteria for pluripotency^18^: this was confirmed by gene expression and immunostaining for the core transcriptional network of pluripotency markers and teratoma formation when introduced into immunocompromised mice (see Methods and Supplementary Figs 2–8). Despite fulfilling criteria for pluripotency and controlling for uniform cell-plating densities, there were marked differences in their differentiation capacities. Specifically, a number of hiPSC lines showed impaired efficiency to differentiate into the endoderm lineage leading us to categorize hiPSC lines as either low or high endodermal differentiation capacity (LDC and HDC, respectively).

### DNA methylation age in pluripotent stem cell lines

We derived methylation levels (*β*, the methylated fraction of cells analysed) for 2,676 autosomal CpH sites (H=A, T, C) at single base-pair resolution using Illumina Infinium HumanMethylation450 BeadChips (‘Illumina 450K BeadChips'). Where multiple DNA extracts from independent cells of the same plate were available, *β*-values were averaged. To ensure the abundant non-CG methylation signal in pluripotent stem cells was not due to incomplete bisulfite conversion, we conducted a number of control experiments (see Methods and Supplementary Figs 9–13). As an additional quality control measure, we computed DNA methylation age (‘DNAm age') using a subset of CpG probes on the Illumina 450K BeadChip^19^. As embryonic stem cells are of prenatal origin, we expect pluripotent cells to exhibit ‘negative age'. Reassuringly, 150 of 161 (93%) pluripotent clones exhibited negative age and none exceeded +0.5 years. There was no significant difference in DNAm age between HDC and LDC pluripotent lines (mean DNAm age: −0.51 (HDC) versus −0.36 (LDC)). DNAm age for the available donor cell lines consistently exceeded 18 years, confirming the adult origin of each donor cell line (Supplementary Fig. 14).

### Promoter DNA methylation in pluripotent stem cells

We also compared promoter methylation of our samples with the ‘Reference Corridor' proposed by Bock *et al*.^9^. Following LiftOver of promoter coordinates from hg18 to hg19, we derived a consensus set of 14,820 promoters where at least one probe of the Illumina 450K BeadChip overlapped; this consensus set of promoters was represented by 109,109 CpG probes. As Reference Corridor estimates were derived using Reduced Representation Bisulfite Sequencing (RRBS), there was, on average, more than four times the number of CpGs assayed per promoter compared with Illumina 450K BeadChips; for this reason we estimated promoter methylation in this cohort of pluripotent stem cells using the median *β*-value, as the median is less sensitive to outliers. However, despite the use of different technologies we found 76.8% of all DNA methylation estimates for this cohort fell within the bounds of the Reference Corridor (Supplementary Fig. 15a). Curiously, the Reference Corridors of 1,734 (11.7%) promoters failed to capture any corresponding Illumina 450K BeadChip estimates; Reference Corridors for these promoters were characterized by intermediate methylation and were mainly marked by a shift towards unmethylated status in this cohort (Supplementary Fig. 15b). All coefficients of variation (CVs) at each of these promoters were <1 in this cohort, indicating stable methylation status at each promoter. Furthermore, many Illumina 450K BeadChip estimates only narrowly missed being captured in the Reference Corridor: almost a quarter (24%) of methylation estimates were within 10% of a Reference Corridor threshold and nearly half (48%) were <20%—magnitudes possibly attributable to the different technologies being compared.

### Non-CG methylation in pluripotent stem cell lines

**Figure 1:**
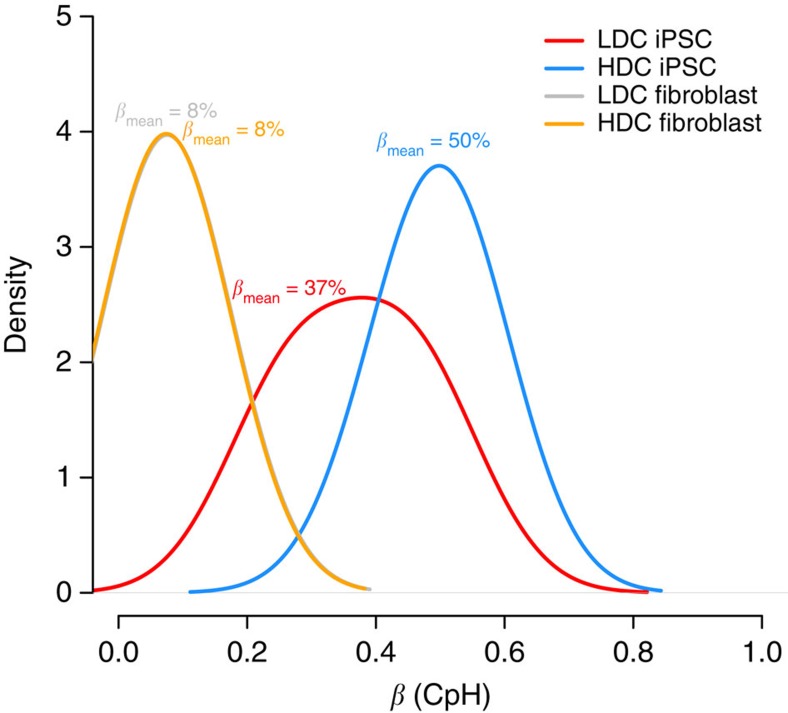
Non-CG methylation is prevalent but reduced in LDC hiPSCs. Density plot illustrating the difference in non-CG methylation profiles between low-differentiation capacity (LDC; red line) and high-differentiation capacity (HDC; blue line) hiPSCs. The difference in mean methylation (Δ*β*) was large (13%) and significant (*P*<7.4 × 10^−4^). For comparison, non-CG profiles are plotted for donor cell lines giving rise to LDC (grey line) and HDC (orange line) hiPSCs; the difference between these donor cell lines was not significant.

To identify methylation variable positions (MVPs) associated with impaired endodermal differentiation capacity, we applied a false discovery rate of 0.05. As previously defined^20^, MVPs are referred to as hyper- or hypo-MVPs when directionality towards differential hyper- or hypomethylation has been ascertained. Nearly all sites tested (2,564 or 96%) were significantly associated and all were hyper-MVPs in HDC hiPSC lines compared with LDC hiPSC lines; a finding not due to technical confounders (Supplementary Fig. 16). Aggregating DNA methylation across all non-CG sites (*β*_mean_), we found LDC and HDC hiPSC lines were characterized by 37% (95% confidence interval (CI)=31–43%) and 50% (95% CI=47–52%) non-CG DNA methylation, respectively. Mean levels of non-CG DNA methylation were not associated with passage number (Pearson's *r*=0.52, *P*=0.28; Supplementary Fig. 17), whereas correlations of non-CG DNA methylation profiles between pluripotent stem cell lines were high (average *r*=0.903) and CVs across cell lines at individual loci were low (average CV=0.49), leading us to infer that the stability of non-CG methylation is actively maintained at this subset of non-CG loci tested. Importantly, the difference in mean non-CG methylation levels between LDC and HDC hiPSC lines (Δ*β*_mean_=13% (95% CI=6–19%); Fig. 1) was significant using both conventional statistical tests (*P*<7.4 × 10^−4^, two sample *t*-test) and permutation testing (empirical *P*=1.5 × 10^−4^). To test whether overall CpG methylation levels were affected in the same way, we performed 100,000 Monte Carlo simulations using 2,676 randomly sampled CpG probes: we found Δ*β*_mean_ between HDC and LDC groups never exceeded 0.005, which intensifies the importance of non-CG DNA methylation loss in LDC hiPSCs. Furthermore, the lack of a global CpG methylation difference between HDC and LDC groups was observed when solely methylated (that is, *β*>80%) CpG probes were randomly sampled, which more accurately reflects the true genomic distribution of CpG methylation. Lastly, the direction and magnitude of the non-CG effect was independent of 6-bp sequence motif surrounding the non-CG locus (Supplementary Fig. 18).

### Gene expression of key (de)methylation genes

We next investigated the effect of DNA (de)methylation machinery on non-CG DNA methylation levels. We profiled gene expression of *DNMT1*, *DNMT3A*, *DNMT3B* and *DNMT3L*, as well as *TET1* by quantitative reverse transcriptase–PCR (qRT–PCR) in the discovery cohort. Of these genes, only *DNMT3B* showed significantly different gene expression between LDC and HDC hiPSC lines (Supplementary Fig. 19a). We also analysed expression of the *DNMT* genes and *TET1* in different passages of the same cell line (FF-iPSC-832-44-R) and found it was positively correlated (*r*=0.901–0.984), although least strongly in *DNMT3B* (*r*=0.825) (Supplementary Fig. 19b). However, there were no significant differences in CpG methylation between LDC and HDC hiPSCs at the promoters of these genes (Supplementary Fig. 20).

### Epigenetic memory analyses

To confirm that non-CG methylation differences between LDC and HDC hiPSCs was not driven by epigenetic memory; for example, predicated by non-CG methylation differences at the donor cell level, we focused our analysis on hiPSCs with matched donor cells. First, donor cells giving rise to both LDC and HDC hiPSC lines (subsequently referred to as ‘LDC donors' and ‘HDC donors') were characterized by notably low and equal levels of non-CG methylation (*β*_mean_: 8% (95% CI=6–10%); Δ*β*_mean_=NS). Non-CG methylation in donors is not at or close to 0%, because the dynamic range of the array is reduced rather than issues of incomplete bisulfite conversion. Second, there were no significant MVPs between LDC and HDC donors. Third, Δ*β*_mean_ between donors and hiPSC lines were notably higher for the HDC group compared with the LDC group (Δ*β*_mean_: 42% versus 29%, respectively), indicating that LDC hiPSCs are more refractory to the acquisition of non-CG methylation (Supplementary Fig. 21). In summary, LDC hiPSCs are characterized by hypomethylation at the majority of non-CG sites assayed on the Illumina 450K BeadChip, which is not predicted by the baseline non-CG profile of the donor cell.

### Polymorphism analyses

We next investigated whether the observed differences in non-CG methylation between LDC and HDC hiPSCs were due to the presence of polymorphisms. We mapped all non-CG sites to data from the 1,000 Genomes Project^21^ and found evidence of genetic variation at just four (0.1%) loci, which confirms that a reduction in non-CG methylation in LDC samples is not due to the loss of methylatable cytosines. We next searched for polymorphisms in the entire probe and found only 61 (2.3%) non-CG probes were potentially affected by polymorphism within 10 bp of the 3′-end of the probe, which rules out confounding by technical factors^22^.

### Non-CG methylation as a biomarker

**Figure 2:**
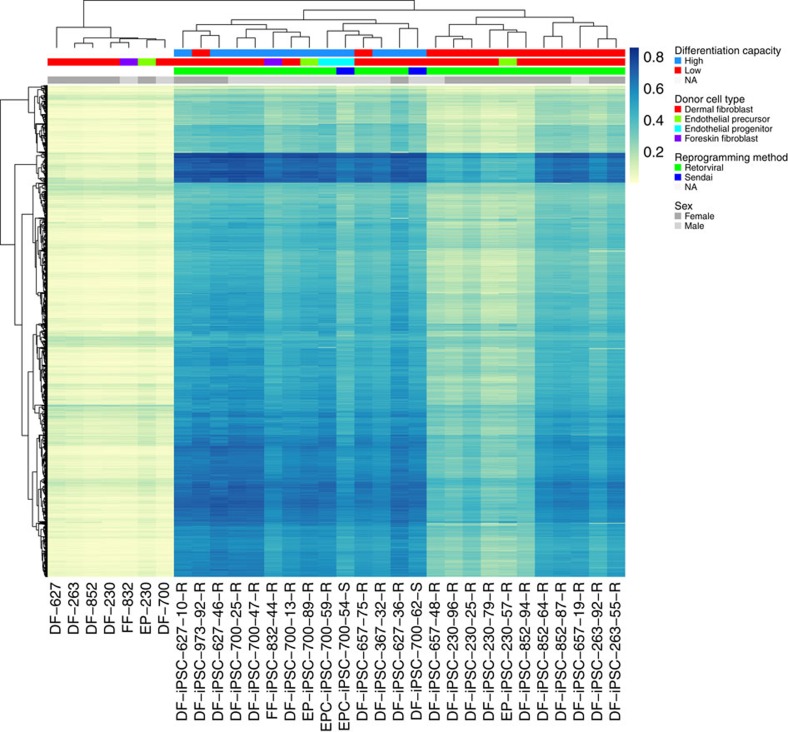
Non-CG DNA methylation profiles can separate LDC from HDC hiPSCs. Unsupervised hierarchical clustering using non-CG methylation data revealed distinct separation of LDC from HDC hiPSCs, as well as from donor cell lines (top annotation bar). The heatmap shows the range of non-CG *β*-values for each sample and illustrates the tendency towards hypermethylation in HDC hiPSCs compared with that in LDC hiPSCs; donor cell lines are characterized by distinctly unmethylated non-CG domains.

Unsupervised hierarchical clustering using all 2,564 significant non-CG probes revealed a largely unequivocal separation of LDC, HDC and donor cell lines (Fig. 2 and Supplementary Fig. 22). Consistent with previous findings^9^,^23^, donor cells and hiPSCs formed separate clades, in our case, illustrating a clear influence of non-CG methylation in the pluripotency phenotype. However, more profound was the correct classification of hiPSC lines (23 of 25, or 92%) into LDC and HDC clades.

**Figure 3:**
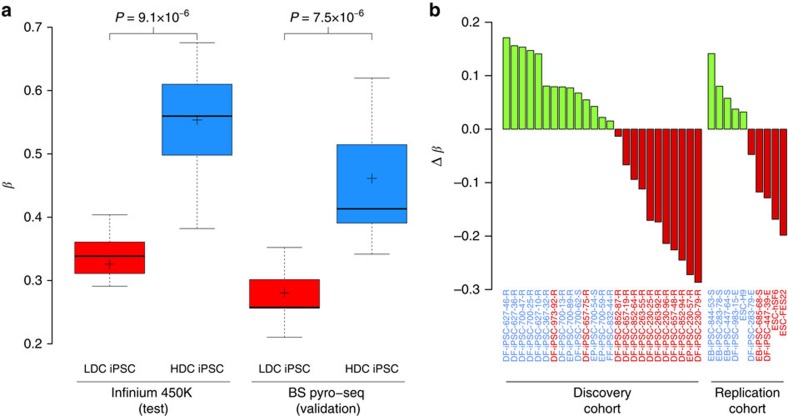
Translation and simplification of a discriminative non-CG assay. We selected 9 of the top 200 non-CG MVPs and aggregated methylation levels for the two hiPSC phenotypes. (**a**) Separation between the two hiPSC phenotypes was maintained and independently validated with bisulfite pyrosequencing. Mean methylation levels are indicated by cross-hairs; *P*-values derived by two sample *t*-tests. (**b**) Sample-specific deviations from an endodermal differentiation capacity threshold in the discovery and replication cohorts. The endodermal differentiation capacity threshold (*β*_mean_=45.8%) is defined as the midpoint between the upper (LDC) and lower (HDC) 95% CIs of mean non-CG levels in the discovery cohort for the nine-probe assay. This threshold correctly identifies 23 of 25 hiPSC lines in the discovery cohort; this finding was replicated in 3 hESC lines and 7 hiPSC lines derived from additional donor cell types and additional reprogramming methods from additional labs. Green bars, HDC (predicted); red bars, LDC (predicted); blue sample labels, HDC (phenotyped); red sample labels, LDC (phenotyped).

For validation and translation into a more simplified assay, we selected nine highly significant MVPs and subjected them to bisulfite pyrosequencing. Methylation levels obtained using this independent platform showed high agreement with the Illumina 450K BeadChip (Pearson's *r*=0.92, *P*=2.2 × 10^−16^; Supplementary Fig. 23) and confirmed significant association of each tested MVP (Supplementary Table 2). Crucially, this subset of MVPs maintains separation between LDC and HDC hiPSC lines, irrespective of analysis platform (Illumina: Δ*β*_mean_=23% (95% CI=15–31%, *P*<9.1.0 × 10^−6^); bisulfite pyrosequencing Δ*β*_mean_=18% (95% CI=12–24%, *P*<7.5 × 10^−6^): two sample *t*-test; Fig. 3a).

The predictive utility of the nine-probe non-CG methylation assay to classify HDC and LDC status was borne out using a replication cohort of ten additional pluripotent stem cell lines comprising three hESC lines and seven hiPSC lines derived from additional donor cell types (erythroblasts) and additional reprogramming methods (episomal vector), grown in an independent laboratory (see Supplementary Table 1). *β*-Values for the nine-probe assay were derived from Illumina 450K BeadChips, to ascertain the mean non-CG methylation for each pluripotent stem cell line and thereby predict endodermal differentiation capacity status; endodermal differentiation capacity phenotying was performed independently and blindly. We defined the threshold for endodermal differentiation capacity status (*β*_mean_=45.8%) as the midpoint between LDC upper (*β*=40%) and HDC lower (*β*=52%) 95% CIs of the mean non-CG level of the nine-probe assay in the discovery cohort. Using this threshold, we correctly identified the endodermal differentiation capacity for nine out of ten (90%) pluripotent stem cell lines (Fig. 3b). In summary, non-CG methylation is a biomarker for stem cell endodermal differentiation capacity, independent of reprogramming method, donor cell type and source of pluripotent cell type (hiPSC and hESC).

## Discussion

The derivation of hiPSCs from easily accessible tissue sources has far reaching implications for the fields of drug screening and development, as well as regenerative medicine^24^. However, a number of challenges remain^25^. One pressing issue is the identification of molecular surrogates that predict the differentiation capacity of an hiPSC to form all three embryonic germ layers^26^. Although variability in differentiation capacity has been attributed to tissue of origin effects^9^,^23^,^27^ and reprogramming technologies^28^, variability still remains even when these variables are kept constant. Although our results are not immune to some degree of overfitting (our study design used more samples in the discovery cohort than the replication cohort), we do show that levels of DNA methylation at a subset of non-CG dinucleotides can serve as a powerful unidirectional biomarker capable of distinguishing pluripotent lines of high and low endodermal differentiation capacity.

Whether this finding extends to predict differentiation capacity in other lineages remains an important question. We tried exploring non-CG DNA methylation in eight pluripotent cell lines (four ES lines and four hiPSC lines) with RRBS data^4^ and corresponding lineage differentiation capacity information^9^; however, we were unable to establish a positive correlation, presumably because of insufficient overlap between 450K- and RRBS-derived non-CG data. Only ten non-CG loci overlap, none of which encompass the biomarker proposed here. This is important because genomic localization matters: although *de novo* methyltransferase activity in pluripotent stem cells is widespread, it has been proposed to be recruited to specific loci, in particular those exhibiting high levels of methylation^4^; of particular note, the non-CG loci assayed on the Illumina 450K array are enriched for regions of H3K36me3 compared with the RRBS loci. Furthermore, unlike Ziller *et al*.^4^ who found only modest correlations (average *r*=0.35) between pluripotent stem cell lines and high CVs at non-CG loci (average CV=3.0), we found high correlations (average *r*=0.903) and low CVs (average CV=0.49)—values that are more akin to those measured at CpGs^4^. It is therefore feasible that the loci assayed in this study represent putatively more functional non-CG targets with respect to differentiation capacity. Second, the different phenotyping methods employed in Bock *et al*.^9^ further complicates a direct comparison with our study.

The functional consequence of non-CG methylation is currently unclear but its presence can directly inhibit the binding of specificity protein 1 (Sp1)^29^. Sp1 contributes to the maintenance of methylation-free CpG islands^30^ and can increase promoter activity^31^, as well as regulating a variety of cellular processes including tissue-specific transcriptional activity^32^, cell differentiation and chromatin remodelling^33^. Experiments carried out by Clark *et al*.^29^ have shown that methylation of the internal CpG site at the consensus Sp1-binding site core (5′- CCGCCC -3′) was able to inhibit Sp1 binding by up to 20% and when the 5′-non-CG site was methylated, Sp1 binding was inhibited by up to 40%. However, when both sites were methylated, Sp1 binding was virtually blocked (95% inhibition)^29^. In the case of Sp1, it is therefore feasible that non-CG methylation may act as a pluripotent stem cell-specific mechanism to keep some constitutively unmethylated genomic regions (for example, CpG islands) methylated.

Although the mechanistic actions of non-CG DNA methylation on differentiation are unknown, our results offer a novel, fast and economic approach for large-scale assessment of hiPSC lines for clinical utility and therapeutic applications. However, future work should include data mining for other putative regulatory sites affected by non-CG DNA methylation and testing the functional effect of occluded DNA-binding proteins in quantitative knockdown paradigms to simulate the intermediately methylated states seen at non-CG loci in pluripotent stem cells.

## Methods

### Cell lines

A total of 32 hiPSC lines, 3 hES cell lines and 7 primary fibroblast cell lines were included in the study (Supplementary Table 1). All hiPSC lines used in the study were either derived from fibroblasts obtained from the Coriell Biodepositiory or derived from tissues listed in Supplementary Table 1 obtained from patients who provided written signed consent ethics approval from the Hertfordshire Ethical Committee (08/H0311/201). All cell lines were negative for mycoplasma contamination.

### qRT–PCR analysis for cell phenotyping

Total RNA was obtained using GenElute Mammalian Total RNA Miniprep Kit (Sigma Aldrich) according to manufacturer's recommendation, followed by complementary DNA synthesis using standard protocols. Briefly, cDNA was synthesized using Superscript II Reverse Transcriptase (Invitrogen) kit with 0.5 μg of total RNA input. qRT–PCR was performed using Sensi Mix Sybr Low Rox Kit (Bioline) then denatured at 94 °C for 5 min and cycled at 94 °C for 30 s, 60 °C for 30 s and 72 °C for 30 s for 40 cycles, followed by a final extension at 72 °C for 10 min, which was performed using a Stratagene Mx3005P thermal cycler machine. qRT–PCR results were normalized to porphobilinogen deaminase. Primer sequences are presented in Supplementary Table 3.

### Immunostaining

hESCs, hiPSCs and their differentiated derivatives were fixed for 20 min at 4 °C in 4% paraformaldehyde and then washed three times in PBS. Cells were then incubated for 20 min at room temperature in PBS containing 10% donkey serum (Serotec) and 0.1% Triton-X (Sigma). Cells were subsequently incubated overnight at 4 °C with the following primary antibodies diluted in 1% donkey serum in PBS (OCT4 1:100 (Santa Cruz; P/N: SC-8628X), NANOG, SOX2, SOX1, SOX17, FOXA2 1:100 (R&D Biosystems); P/Ns: AF1997, AF2018, AF3369, AF1924 and AF2400, respectively)). Cells were then washed three times in PBS and incubated with Texas Red or fluorescein isothiocyanate-conjugated Donkey anti-mouse IgG (1:1,000, Invitrogen; P/N: A21202) or Donkey anti-rabbit IgG (1:1,000, Invitrogen; P/N: A10042), or Donkey anti-goat IgG (1:1,000, Invitrogen; P/N: A11057) for 2 h at room temperature. The cells were washed three times in PBS (with Hoechst, Sigma-Aldrich, 1:10,000 in the first wash to stain the nuclei blue). Images were obtained with Zeiss Axiovert 200M microscope with AxioVision Rel 4.7 software (Carl Zeiss, Jena, Germany).

### Flow cytometry

Cells were washed twice in PBS and then incubated for 20 min at 37 °C with cell dissociation buffer (Invitrogen). Cells were dissociated by gentle pipetting and resuspended at ∼0.1–1 × 10^5^ cells per ml in PBS plus 3% normal goat serum containing 0.1% azide (Serotec). Cells were then fixed for 20 min at 4 °C in 4% paraformaldehyde and then washed three times in PBS. Cells were then incubated for 2 h at room temperature with primary antibody (OCT4 (1:100; Santa Cruz; P/N: SC-8628X), CXCR4 (1:100; R&D Biosystems; P/N: MAB173) and SOX17 (1:100; R&D Biosystems; P/N: AF1924)) or mouse IgG isotype control (1:100; BD Biosciences-Pharmigen; P/N: 555749). Cells were then washed three times in PBS+3% normal goat serum containing 0.1% azide and then incubated with secondary antibodies for 2 h at room temperature. Unbound secondary antibody was removed by three washes in PBS. Cells were analysed in a Beckman Coulter CyAn ADP flow cytometer (Beckman Coulter, High Wycombe, UK).

### Teratoma assay

hiPSCs were harvested mechanically immediately before implantation, and ∼1 × 10^5^ cells were inoculated beneath the testicular capsule of 6- to 8-week-old C.B.−17/GbmsTac-scid-bgDF N7 male mice (Taconic M&B) housed and maintained at 20–24 °C, 50% room humidity, in a 14-h light and 10-h dark cycle with food and water *ad libitum*. The mice were killed after 60 days and the injected testes were cut into equal pieces using a razor blade. The material was fixed overnight in 4% neutral buffered formaldehyde and dehydrated through a graded series of alcohols to xylene. The tissue was embedded in paraffin and serially sectioned at 5 μm, followed by haematoxylin and eosin staining and characterization. A human origin of the selected areas was verified by fluorescent *in situ* hybridization (human-specific probes, CEP XY; Vysis Inc.). The experiments were performed with permission from The Stockholm North Ethical Committee on Animal Experiments (Stockholm, Sweden; permission number: N105/07).

### *In vitro* differentiation

Endoderm differentiation has been described as follows^34^. Briefly, hESCs and hiPSCs were harvested with dispase (1 mg ml^−1^) for 1 h and then seeded in gelatinized fetal bovine serum-coated plates in chemically defined media (CDM) supplemented with Activin A and fibroblast growth factor 2 (FGF2) for 24 h. To obtain endodermal progenitors, cells were grown in CDM with Polyvinyl Alcohol supplemented with Activin A (100 ng ml^−1^), fibroblast growth factor 2 (FGF2) (20 ng ml^−1^), bone morphogenetic factor 4 (BMP4) (10 ng ml^−1^) and LY294002 (10 mM) for 3 days. For neuroectoderm differentiation, cells were grown in CDM with polyvinyl alcohol supplemented with SB431542 (10 μM), FGF2 (12 ng ml^−1^) and Noggin (200 ng ml^−1^) for 10 days. For BMP4 treatment, cells were grown in CDM with bovine serum albumin supplemented with BMP4 (10 ng ml^−1^) and SB431542 (10 μM) for 10 days. For pancreatic differentiation, human pluripotent stem cells were differentiated into endoderm using CDM supplemented with Activin A (100 ng ml^−1^), BMP4 (10 ng ml^−1^; R&D Systems), basic fibroblast growth factor (20 ng ml^−1^) and LY294002 (10 μM; Promega) for 3 days. After definitive endoderm-differentiation stage, cells were cultured in Advanced DMEM supplemented with BSA, SB-431542 (10 μM; Tocris), fibroblast growth factor 10 (FGF10) (50 ng ml^−1^; AutogenBioclear), all-*trans* retinoid acid (2 μM; Sigma) and Noggin (150 ng ml^−1^; R&D Systems) for 3 days. After that, cells were cultured in Advanced DMEM+human FGF10 (50 ng ml^−1^; AutogenBioclear), all-*trans* retinoid acid (2 μM; Sigma), KAAD-cyclopamine (0.25 M; Toronto Research Chemicals) and Noggin (150 ng ml^−1^; R&D Systems) for 3 days. Finally, the cells were cultured in human KGF (50 ng ml^−1^; R&D Systems) for 3 days. For maturation of pancreatic progenitors, cells were grown in Advanced DMEM+1% vol/vol B27 and DAPT (1 mM) for 3 days and for 3 additional days in Advanced DMEM+1% vol/vol B27. More details can be found in ref 35.

### Confirmation of pluripotency

Pluripotency was assessed in a number of ways. First, we analysed activity of the core pluripotency markers (*OCT4, NANOG* and *SOX2* for hiPSCs in the discovery cohort and hESCs in the replication cohort, and *OCT4* only for hiPSCs in the replication cohort) in undifferentiated human pluripotent stem cells (hPSCs) using qRT–PCR; results are presented in Supplementary Fig. 2a,b. Second, we verified the absence of reprogramming transgenes by endogenous and exogenous gene expression analysis by qRT–PCR of *OCT4, SOX2, KLF4* and *cMYC*; results for the discovery cohort are presented in Supplementary Fig. 3. Third, we confirmed homogeneity of cells in a selection of discovery cohort hiPSCs by immunofluorescence of OCT4, NANOG and SOX2 (Supplementary Fig. 4a), and flow cytometry of OCT4 (Supplementary Fig. 4b). Fourth, we assessed the ability of differentiated hPSCs to produce progenitors of three germ layers. Gene expression analysis of SOX2 and SOX1 (neuroectoderm), and SOX7 and HAND1 (primitive endoderm and extraembryonic ectoderm markers) was performed in the discovery cohort; results are presented in Supplementary Fig. 5a,b. Immunostaining for SOX2 and SOX1 was performed for a selection of the discovery cohort; results are presented in Supplementary Fig. 5c.

### Endodermal differentiation capacity scoring

We categorized hPSCs as HDC if they generated endodermal cells expressing the definitive endoderm markers *SOX17, FOXA2* and *CXCR4*, and the primitive streak markers *EOMES* and *MIXL1*. For the hiPSCs in the discovery cohort and hESCs in the replication cohort, we used qRT–PCR for gene expression analysis of *SOX17, FOXA2, EOMES* and *MIXL1* (Supplementary Fig. 6a). For the hiPSCs in the replication cohort, we used qRT–PCR for gene expression analysis of *SOX17* and *CXCR4* (Supplementary Fig. 6b). For a selection of hiPSCs from the discovery cohort, we used immunostaining for SOX17, FOXA2 and EOMES (Supplementary Fig. 6c), and flow cytometry for CXCR4 (Supplementary Fig. 6d). For the replication cohort, we used flow cytometry for SOX17 and CXCR4 (Supplementary Fig. 6e). Unlike HDC lines, LDC lines showed decreased expression of the endodermal markers, generated a low yield of SOX17- and FOXA2-positive cells, and exhibited low yields of CXCR4- and/or SOX17-expressing cells.

### Characterization of differentiation capacity

To further study the limited capacity to differentiate into endoderm, five LDC and one HDC hiPSC lines were induced to generate pancreatic progenitors using a combination of retinoic acid, *NOGGIN*, *FGF10* and inhibitor of NODAL signalling. We measured the expression of *PDX1* (a transcription factor that is expressed during pancreatic development) and hormonal markers such as Glucagon and Insulin, after 18 days of differentiation. We found that these genes were not upregulated in LDC lines, whereas the expression levels were high in the HDC line consistent with LDC lines being refractory to endodermal differentiation (Supplementary Fig. 7a–c). We also performed teratoma assays on 2 LDC and 1 HDC hiPSC line to confirm a quantitative reduction in the yield of endodermal progenitors in LDC hiPSCs compared with HDC hiPSCs (see ref. 36 and Supplementary Table 4). The final status of endodermal differentiation capacity status for each pluripotent cell line is presented in Supplementary Table 1. In summary, hPSC lines were categorized as LDC if they produced <50% Sox17- or CXCR4-expressing cells as revealed by FACS; HDC hPSC lines produced >60% expressing cells.

### DNA preparation

Genomic DNA was extracted from cells using GenElute Mammalian DNA Miniprep Kit (Sigma) or AllPrep DNA/RNA Mini Kit (Qiagen) according to the manufacturer's protocol. DNA purity and quantity were checked by spectrophotometry (NanoDrop ND-1000, Thermo Scientific); DNA quality (fragment integrity) was assessed using 1% tris-borate-EDTA agarose gels. Before bisulfite conversion, DNA concentration was estimated by fluorometry (Qubit 2.0, Life Technologies) and 1 μg DNA was bisulfite converted (EZ DNA Methylation Kit, Zymo Research) in accordance with the manufacturer's protocol with alternative incubation conditions (that is, 16 × cycles (95 °C for 30 s, 50 °C for 60 min)).

### DNA methylation profiling

Following bisulfite conversion quality control with quantitative PCR (qPCR; see below), bisulfite-converted DNA extracts were hybridized to Infinium 450K BeadChips (Illumina) and scanned with iScan (Illumina) in accordance with the manufacturer's protocol. Following scanning, raw data in the form of IDAT files were funnelled through the *ChAMP* analysis pipeline^37^ implemented in R (http://www.R-project.org/). *ChAMP* is an integrated workflow that ues a number of Bioconductor packages. Within *ChAMP*, IDAT files were loaded and normalized using *minfi*^38^. Probes with low detection metrics (*P*<0.01) in at least one DNA extract were excluded. Raw (non-normalized) methylation levels (*β*, the methylated fraction of cells assayed) were calculated (Signal_Meth_/(Signal_Meth_+Signal_Unmeth_)) for each DNA extract in the discovery and replication cohorts (*N=*177+20). For samples with multiple technical replicates, Euclidian distances between DNA extracts based on *β* were plotted in two-dimensional space for single-nucleotide polymorphism (rs#) probes; two outliers that misclustered were identified by observation with the naked eye and were removed from further analysis. For the remaining DNA extracts (*N*=195), signal intensities for all probes (including 834 quality control probes used to assess bisulfite conversion, extension, hybridization, negative controls, specificity, staining and target removal) were normalized using the subset-quantile within array normalization SWAN^39^ method. Data from the quality control probes were combined with coded systematic information (batch, array position and slide) and phenotypic information (sex, cell type and cell line) for singular value decomposition^40^ analysis to identify confounding factors. Singular value decomposition analysis indicated that substantial components of DNA methylation were correlated with biological factors but not with technical factors (Supplementary Fig. 12). All empirical analyses were performed using normalized *β*-values with the exception of statistical testing, to identify methylation variable positions (MVPs); here, *β*-values were logit transformed into *M*-values to reduce heteroscedascity^41^. Differences in *M*-values were tested for statistical significance using moderated *t*-tests where sample variances were shrunk by computing empirical Bayes posterior means using the *limma* package^42^, with multiple DNA extracts appropriately specified. As samples contained a mixture of males and females, we omitted probes mapping to chromosomes X and Y.

### Bisulfite conversion quantity control

We controlled bisulfite conversion efficiency using five separate analyses. First, we used qPCR to amplify eight randomly selected samples and four controls using two primer pairs (one for converted DNA and the other for unconverted DNA; see Supplementary Fig. 5). Desulfonated and purified products were quality controlled for bisulfite conversion using triplicate qPCR reactions in a final reaction volume of 12.5 μl (6.25 μl reaction buffer (MESA Blue, Eurogentec), 0.625 μl primer pair (10 μM each; see below), 4.375 μl water and 1.25 μl DNA). qPCR conditions were as follows: 95 °C for 5 min, 40 × cycles (95 °C for 15 s, 60 °C for 60 s), meltcurve). Primers were designed for converted (F: 5′- TGGTGATGGAGGAGGTTTAGTAAGT -3′, R: 5′- AACCAATAAAACCTACTCCTCCCTTAA -3′) and unconverted (F: 5′- TGGTGATGGAGGAGGCTCAGCAAGT -3′, R: 5′- AGCCAATGGGACCTGCTCCTCCCTTGA -3′) DNA. Second, we manually inspected *β*-plots. Capitalizing on the Illumina 450K BeadChip's probe design, we reasoned that incomplete bisulfite conversion would not only artificially inflate non-CG estimates but would also disrupt the distribution (and detection rate) of CpG *β*-values (Supplementary Fig. 6). Third, we made use of the inbuilt Illumina 450K BeadChip control probes. The Illumina 450K BeadChip contains hundreds of control probes measured in each of its red and green channels; from the green channel only, we extracted all 6 Infinium I Assay bisulfite conversion control probe intensities (3 converted (*BIC*_1–3_)+3 unconverted (*BIU*_1–3_)) and the mean of the 613 negative control probe intensities (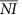), to derive an estimate of bisulfite conversion efficiency (Supplementary Fig. 7). Next, we calculated background-adjusted Infinium I Assay bisulfite conversion control probe intensities:


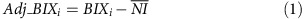


where *X* is control probe design (converted/unconverted) and *i* is the probe number (1–3). Bisulfite conversion efficiency was then calculated as:


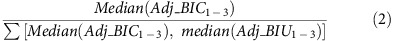


Fourth, to calibrate our bisulfite conversion efficiency estimates we profiled whole-genome-amplified and subsequently *in vitro*-methylated (*SssI* methyltransferase) DNA on the Illumina 450K BeadChip in duplicate (Supplementary Fig. 8). Finally, we tested two additional ultra-stringent bisulfite conversion protocols using the H9 hESC line (Supplementary Fig. 9). As non-CG methylation could be an artefact of incomplete or insufficient bisulfite conversion, it has been suggested that extra steps be performed. To assess the effect of these extra steps we conducted duplicate reactions of the following conditions: (1) standard bisulfite conversion thermal cycling conditions (employed in this study); (2) similar as in (1) +prior proteinase K and two rounds of phenol–chloroform extraction; (3) similar as in (2)+second round of bisulfite conversion.

### Bisulfite pyrosequencing

One microgram of genomic DNA was bisulfite converted (Imprint DNA Modification Kit, Sigma Aldrich) according to the manufacturer's protocol. Purified samples were amplified by PCR carried out in a final reaction volume of 10 μl (250 nM forward and reverse primers (F and R, see Supplementary Table 5), 0.25 U *Taq* (HotStarTaq DNA Polymerase, Qiagen) and 0.2 mM dNTPs). PCR conditions were as follows: 95 °C for 15 min, 40 × cycles (94 °C for 30 s, 48 °C for 30 s, 72 °C for 30 s), 72 °C for 5 min. Single-strand PCR products were purified (PyroMark Q96 Vacuum Prep Workstation, Qiagen). Pyrosequencing was performed on a PyroMark Q96 MD (Qiagen) using PyroMark Gold Q96 Reagents (Qiagen) and locus-specific pyrosequencing (PSQ) primers (see Supplementary Table 5) in accordance with the manufacturer's protocol. The degree of methylation at a non-CG site was determined as a single-nucleotide polymorphism by PyroMark MD software.

### qRT–PCR for *DNMTs* and *TET1* gene expression

Total RNA was extracted from cells using TRI Reagent (Sigma Aldrich). Total RNA (0.5 μg) was reverse transcribed using RevertAid H Minus Reverse Transcriptase (Thermo Scientific). qRT–PCR reaction mixtures were prepared according to the manufacturer's recommendations using LightCycler 480 SYBR Green I Master (Roche). Reactions were performed using LightCycler 480 II (Roche). Standard curves were generated for each primer set and were used to calculate the relative quantity of each primer set by the relative quantity of expression of reference gene. Primers were taken from ref. 43 and are presented in Supplementary Table 6.

### Bioinformatic analysis using published data sets

Bock *et al*.^9^: methylation thresholds and data for the Reference Corridor of 16,383 promoters was downloaded from the Supplementary Information section of the publication. Genome assembly coordinates of these promoters were converted from NCBI/hg18 to GRCh37/hg19 using the LiftOver utility implemented in Galaxy^44^,^45^,^46^. Sixty-five of 16,383 (0.4%) hg18 promoter coordinates could not be mapped to hg19.

Ziller *et al*.^4^: processed and scored non-CG methylation data from eight hPSC lines (hES H1, hES H9, hES HUES1, hES HUES8, hiPS 17b, hiPS 27b, hiPS 27e and hiPS 29e) with Scorecard^9^ data were downloaded from Gene Expression Omnibus (GSE27432). Genome assembly coordinates were converted from NCBI/hg18 to GRCh37/hg19 using the LiftOver utility implemented in Galaxy^44^,^45^,^46^. Depending on the sample, between 107 and 137 non-CG with hg18 loci could not be mapped to hg19.

The intersect of genomic loci/regions between two or more data sets was determined using the *GenomicRanges*^47^ package of Bioconductor and implemented in *R*. Sequences for genomic regions was retrieved using UCSC hg19 from the *BSgenome* package of Bioconductor and implemented in *R*. Sequence matching was achieved using the *Biostrings* package of Bioconductor and implemented in *R*.

## 

## Additional information

**Accession codes:** Methylation microarray data have been deposited in the Gene Expression Omnibus under accession code GSE59091.

**How to cite this article:** Butcher, L. M. *et al*. Non-CG DNA methylation is a biomarker for assessing endodermal differentiation capacity in pluripotent stem cells. *Nat. Commun.* 7:10458 doi: 10.1038/ncomms10458 (2016).

## Supplementary Material

Supplementary Information Supplementary Figures 1-23, Supplementary Tables 1-6 and Supplementary Reference
