# Supplementary material for: Non-CG DNA methylation is a biomarker for assessing endodermal differentiation capacity in pluripotent stem cells
Source: Nat Commun. 2016 Jan 29;7:10458. doi: 10.1038/ncomms10458 (PMC4740175; doi:10.1038/ncomms10458)
Supplement: Supplementary Information — Supplementary Figures 1-23, Supplementary Tables 1-6 and Supplementary Reference [file ncomms10458-s1.pdf]

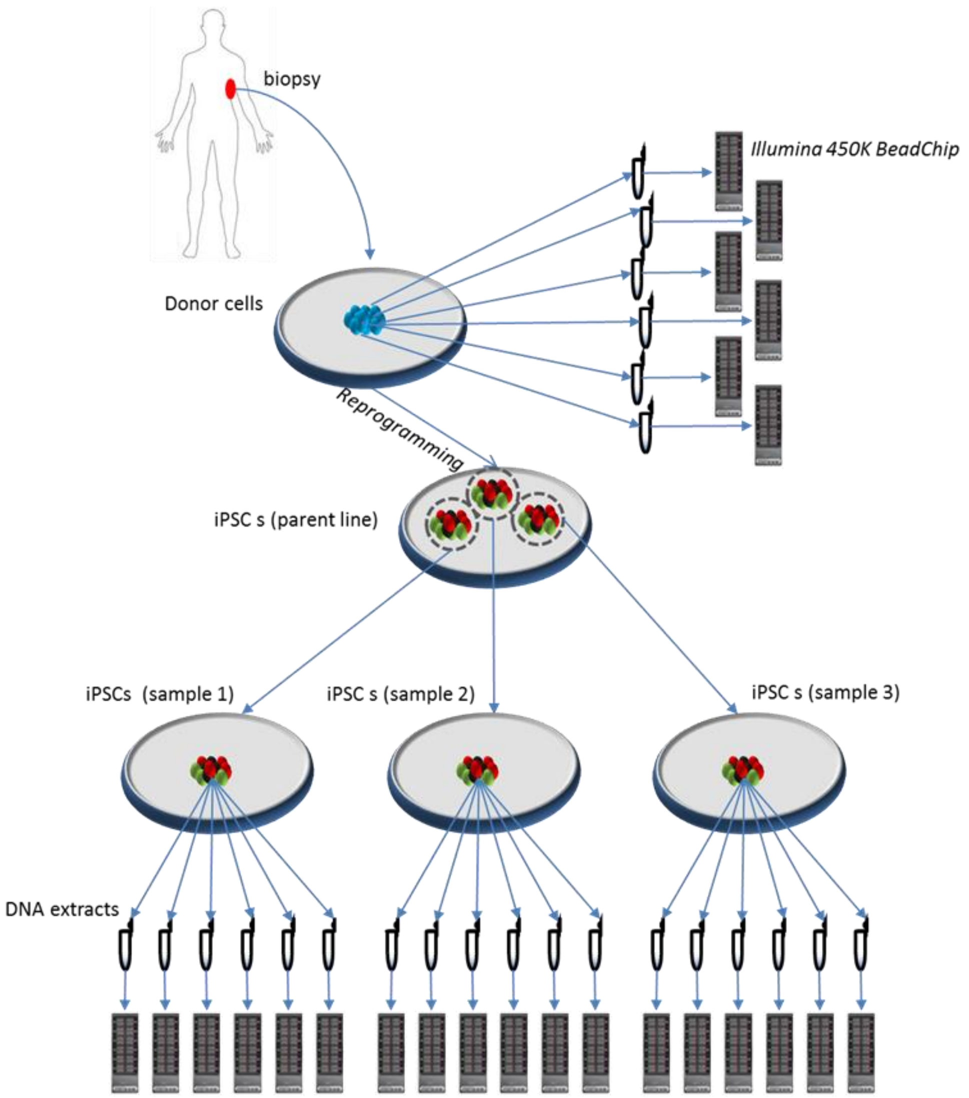

Supplementary Figure 1 Schematic representation of iPSC line derivation and the samples assayed for DNA methylation. Most biological replicates or cell lines (i.e., “samples”) in this study were represented by multiple technical replicates (i.e., “DNA extracts” – independent cells of the same plate); DNA extracts were run on separate Illumina 450K BeadChips and averaged, where appropriate, to provide a consensus “sample” estimate.

a

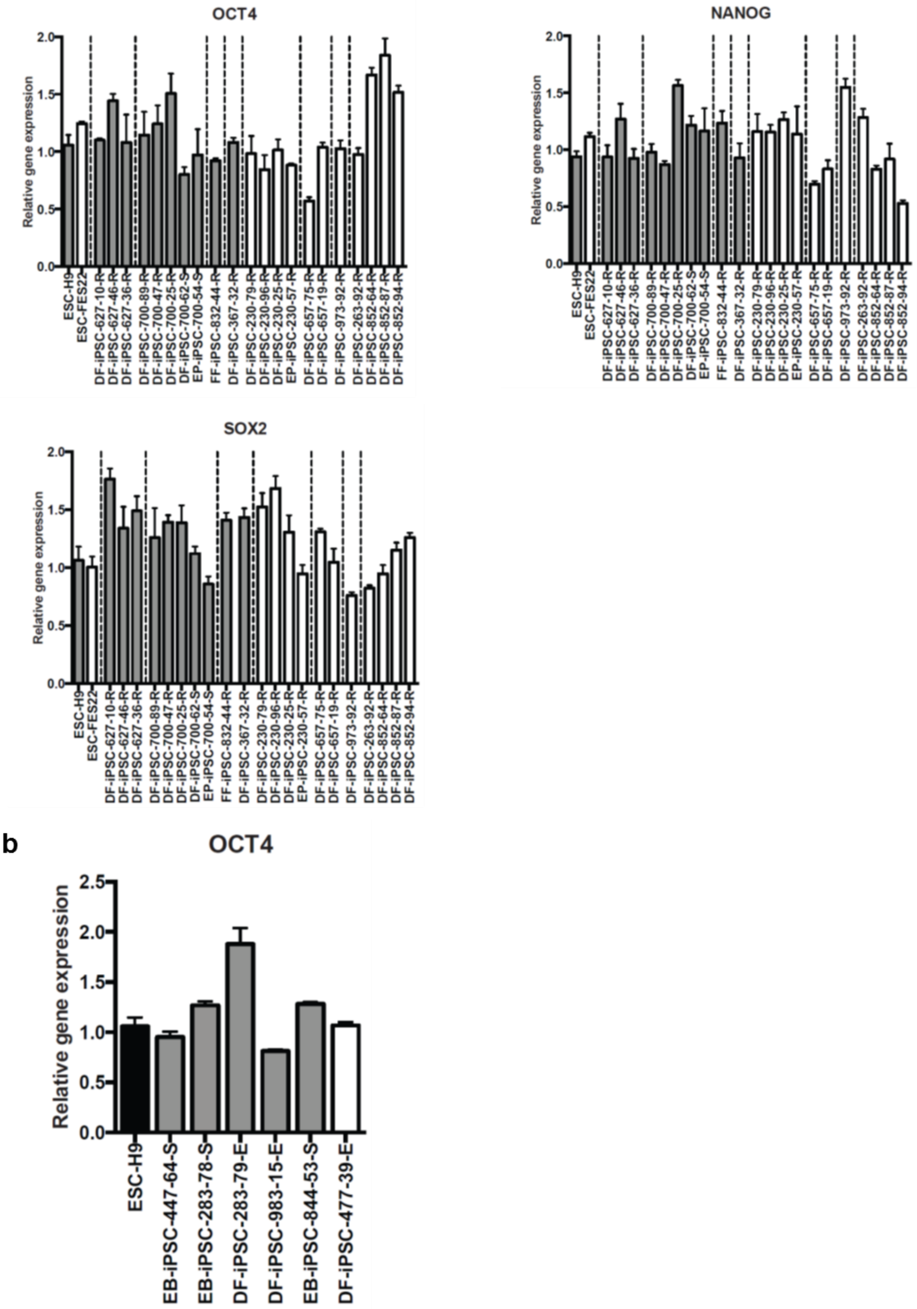

Supplementary Figure 2: Characterisation of pluripotency in hiPSCs. **(a)** qRT-QPCR for OCT4, NANOG and SOX2 in undifferentiated hiPSCs grown on feeders in the presence of knock out serum replacer (KSR) supplemented with FGF2. **(b)** qRT-PCR for OCT4 in the replication cohort. Together these data show that expression of these genes in the hiPSCs is similar to ES cells. Dashed vertical lines divide cell lines derived from different donors. Error bars indicate standard deviations from triplicate experiments.

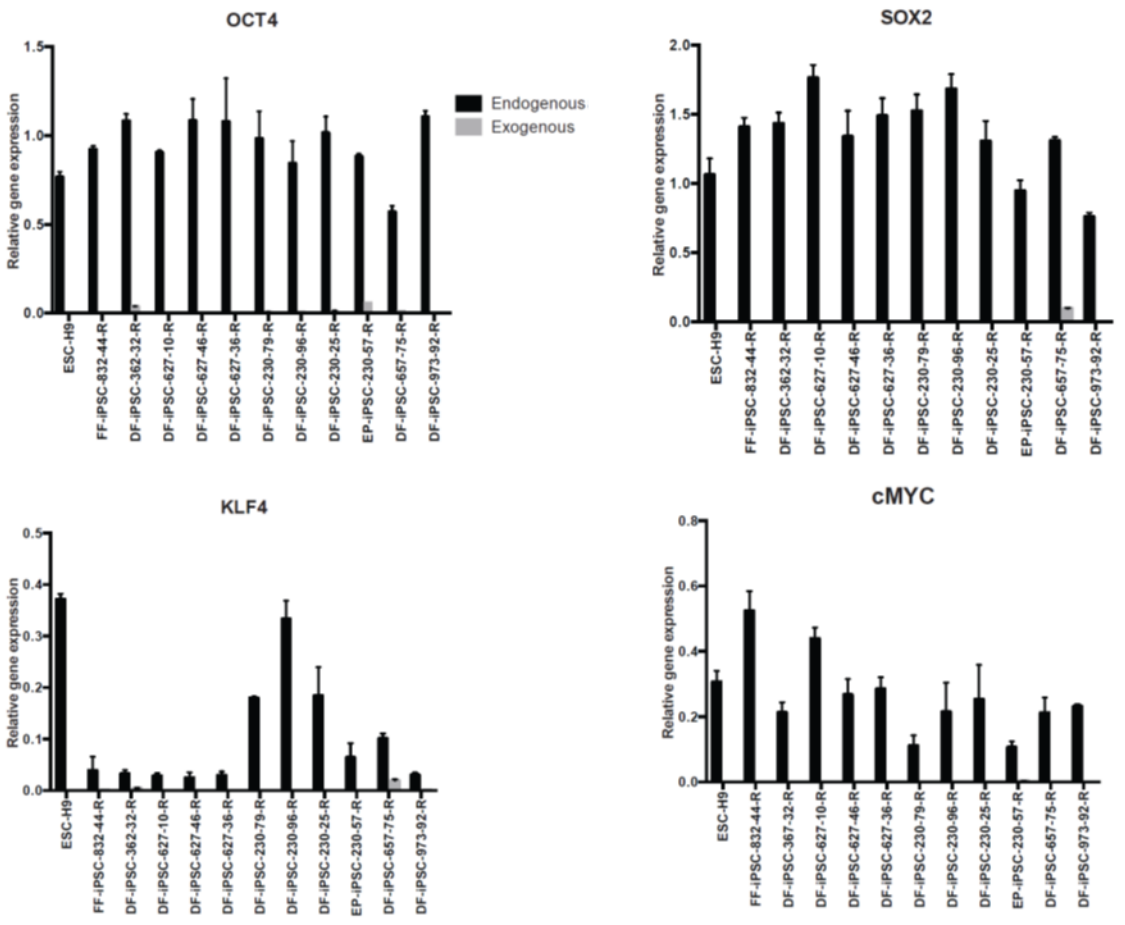

Supplementary Figure 3: Absence of reprogramming transgenes OCT4, SOX2, KLF4 and cMYC by exogenous and endogenous gene expression in hiPSCs used in the discovery cohort. Error bars indicate standard deviations from triplicate experiments.

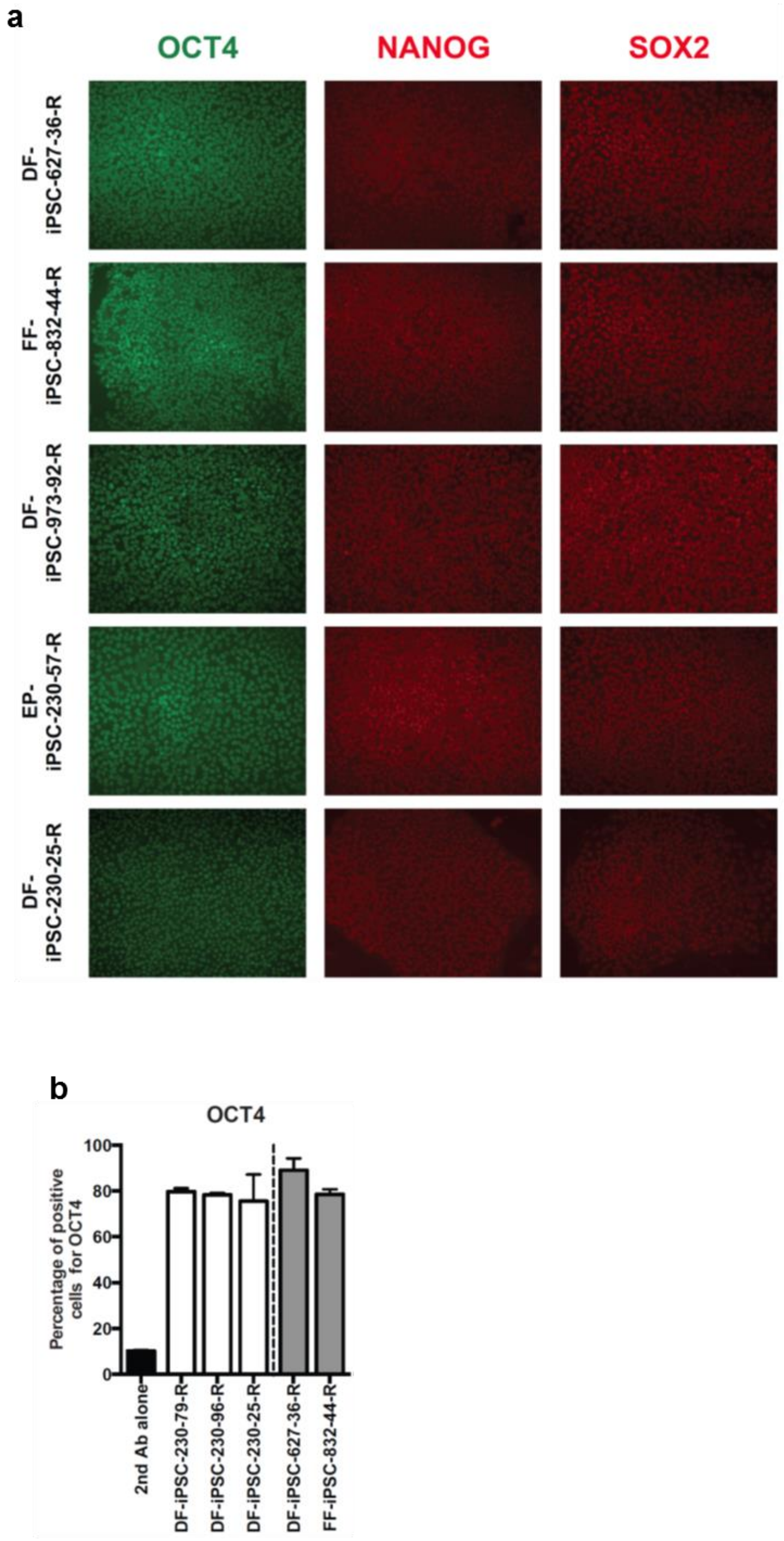

Supplementary Figure 4: Homogeneity of pluripotency in hiPSCs. **(a)** Immunofluorescence staining of hiPSCs shows expression of OCT4 (green), NANOG (red) and SOX2 (red). **(b)** Flow cytometry of the OCT4 protein in the discovery cohort. Error bars indicate standard deviations from triplicate experiments.

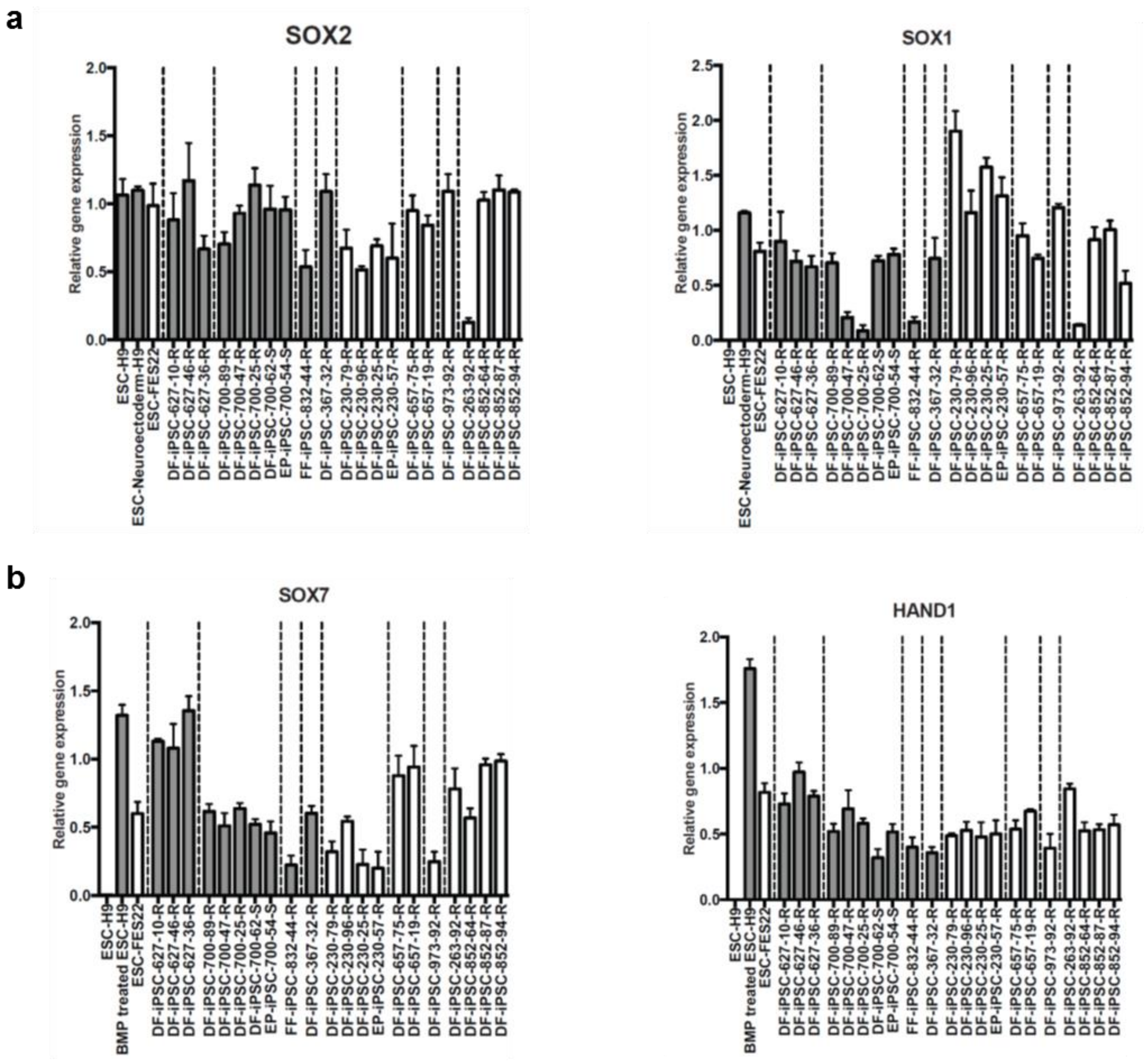
a
SOX2
Relative gene expression
2.0
1.5
1.0
0.5
0.0
ESC-H9
ESC-Neuroectoderm-H9
ESC-FES22
DF-iPSC-627-10-R
DF-iPSC-627-46-R
DF-iPSC-627-36-R
DF-iPSC-700-89-R
DF-iPSC-700-47-R
DF-iPSC-700-25-R
DF-iPSC-700-62-S
EP-iPSC-700-54-S
FF-iPSC-832-44-R
DF-iPSC-367-32-R
DF-iPSC-230-79-R
DF-iPSC-230-96-R
DF-iPSC-230-25-R
EP-iPSC-230-57-R
DF-iPSC-657-75-R
DF-iPSC-657-19-R
DF-iPSC-973-92-R
DF-iPSC-263-92-R
DF-iPSC-852-64-R
DF-iPSC-852-87-R
DF-iPSC-852-94-R
SOX1
Relative gene expression
2.5
2.0
1.5
1.0
0.5
0.0
ESC-H9
ESC-Neuroectoderm-H9
ESC-FES22
DF-iPSC-627-10-R
DF-iPSC-627-46-R
DF-iPSC-627-36-R
DF-iPSC-700-89-R
DF-iPSC-700-47-R
DF-iPSC-700-25-R
DF-iPSC-700-62-S
EP-iPSC-700-54-S
FF-iPSC-832-44-R
DF-iPSC-367-32-R
DF-iPSC-230-79-R
DF-iPSC-230-96-R
DF-iPSC-230-25-R
EP-iPSC-230-57-R
DF-iPSC-657-75-R
DF-iPSC-657-19-R
DF-iPSC-973-92-R
DF-iPSC-263-92-R
DF-iPSC-852-64-R
DF-iPSC-852-87-R
DF-iPSC-852-94-R
b
SOX7
Relative gene expression
2.0
1.5
1.0
0.5
0.0
ESC-H9
BMP treated ESC-H9
ESC-FES22
DF-iPSC-627-10-R
DF-iPSC-627-46-R
DF-iPSC-627-36-R
DF-iPSC-700-89-R
DF-iPSC-700-47-R
DF-iPSC-700-25-R
DF-iPSC-700-62-S
EP-iPSC-700-54-S
FF-iPSC-832-44-R
DF-iPSC-367-32-R
DF-iPSC-230-79-R
DF-iPSC-230-96-R
DF-iPSC-230-25-R
EP-iPSC-230-57-R
DF-iPSC-657-75-R
DF-iPSC-657-19-R
DF-iPSC-973-92-R
DF-iPSC-263-92-R
DF-iPSC-852-64-R
DF-iPSC-852-87-R
DF-iPSC-852-94-R
HAND1
Relative gene expression
2.0
1.5
1.0
0.5
0.0
ESC-H9
BMP treated ESC-H9
ESC-FES22
DF-iPSC-627-10-R
DF-iPSC-627-46-R
DF-iPSC-627-36-R
DF-iPSC-700-89-R
DF-iPSC-700-47-R
DF-iPSC-700-25-R
DF-iPSC-700-62-S
EP-iPSC-700-54-S
FF-iPSC-832-44-R
DF-iPSC-367-32-R
DF-iPSC-230-79-R
DF-iPSC-230-96-R
DF-iPSC-230-25-R
EP-iPSC-230-57-R
DF-iPSC-657-75-R
DF-iPSC-657-19-R
DF-iPSC-973-92-R
DF-iPSC-263-92-R
DF-iPSC-852-64-R
DF-iPSC-852-87-R
DF-iPSC-852-94-R

c

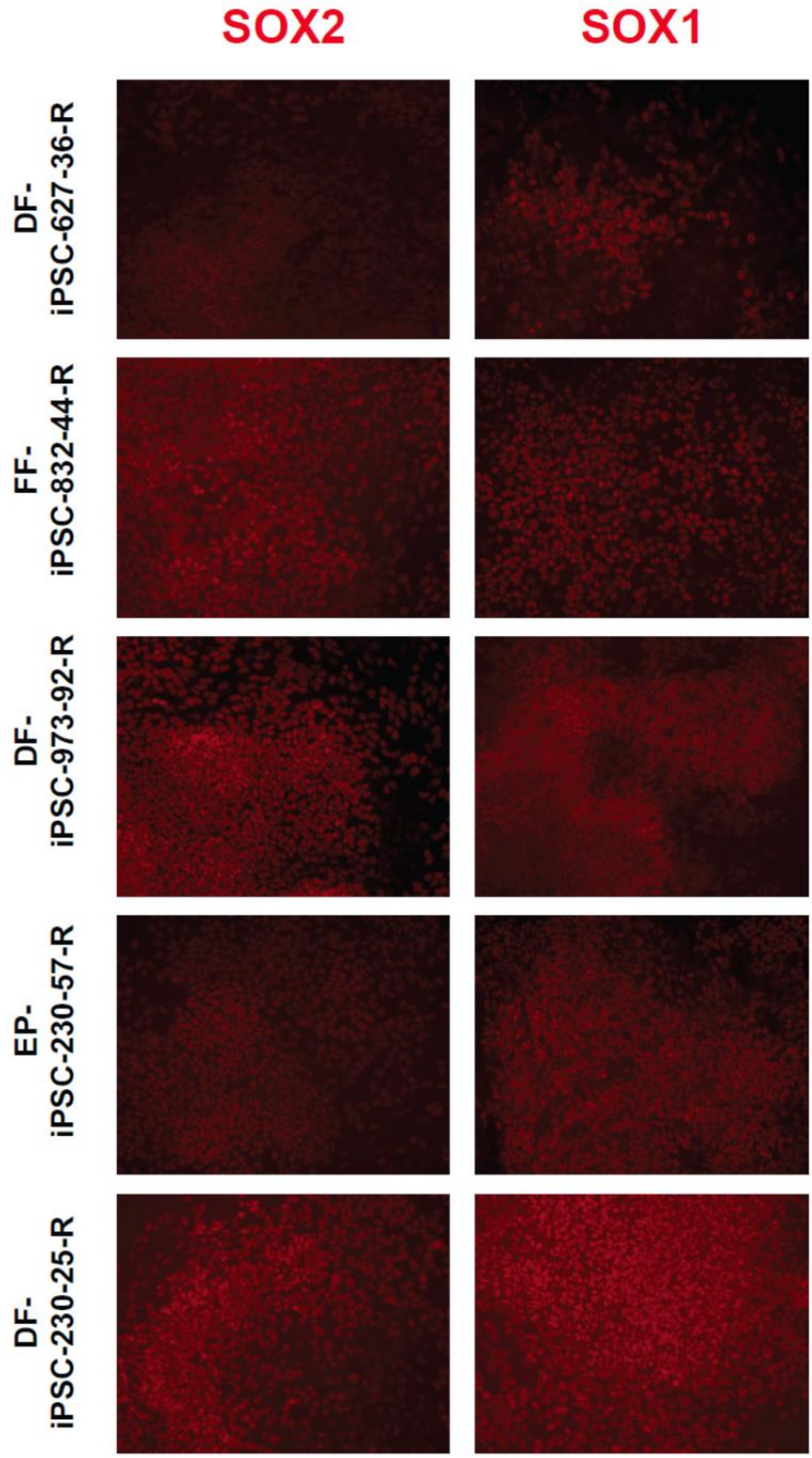
SOX2
SOX1
DF-iPSC-627-36-R
FF-iPSC-832-44-R
DF-iPSC-973-92-R
EP-iPSC-230-57-R
DF-iPSC-230-25-R

Supplementary Figure 5: hiPSCs produce progenitors of all three germ layers. (a) qRT-QPCR and (b) immunofluorescence analyses show that hiPSCs grown for 12 days in chemically defined medium (CDM) supplemented with FGF2 (12 ng/ml), SB431542 (10 μM) and Noggin (200 ng/ml) express neuroectoderm markers (SOX2 and SOX1). (c) BMP-treated hiPSCs express SOX7 (primitive endoderm) and HAND1 (extraembryonic ectoderm). Error bars indicate standard deviations from triplicate experiments.

a

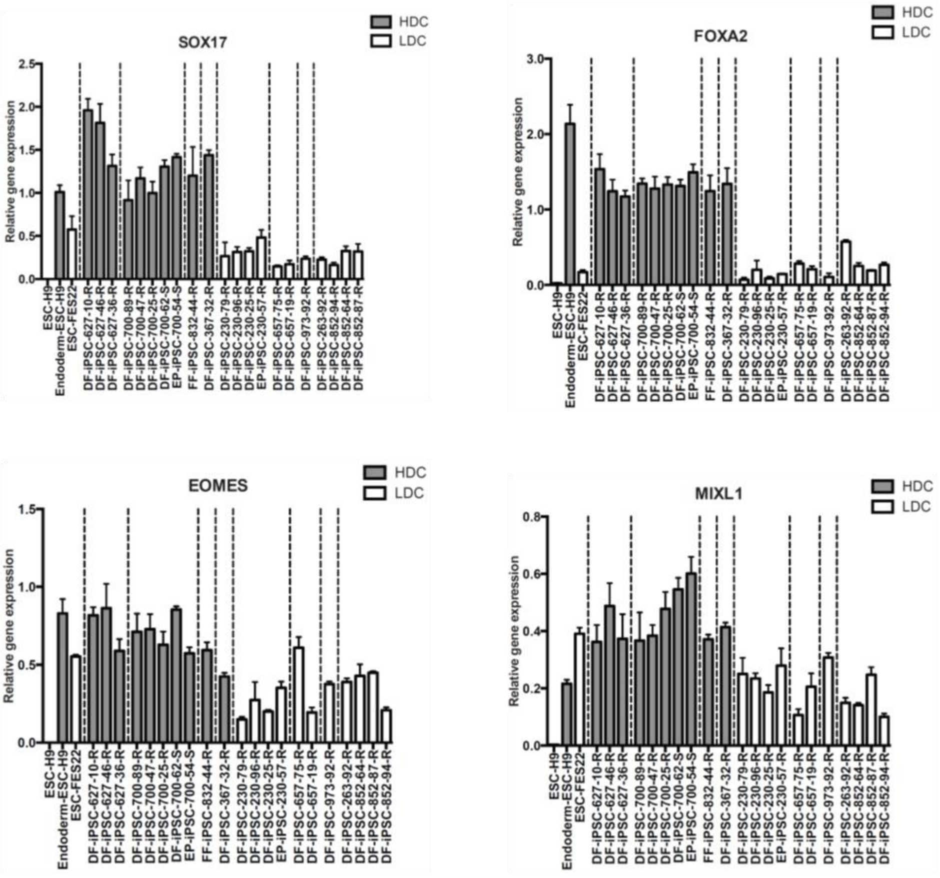
SOX17
FOXA2
EOMES
MIXL1
HDC
LDC
Relative gene expression
ESC-H9
Endoderm-ESC-H9
ESC-FES22
DF-iPSC-627-10-R
DF-iPSC-627-46-R
DF-iPSC-627-36-R
DF-iPSC-700-89-R
DF-iPSC-700-47-R
DF-iPSC-700-25-R
DF-iPSC-700-62-S
EP-iPSC-700-54-S
FF-iPSC-832-44-R
DF-iPSC-367-32-R
DF-iPSC-230-79-R
DF-iPSC-230-96-R
DF-iPSC-230-25-R
EP-iPSC-230-57-R
DF-iPSC-657-75-R
DF-iPSC-657-19-R
DF-iPSC-973-92-R
DF-iPSC-263-92-R
DF-iPSC-852-94-R
DF-iPSC-852-64-R
DF-iPSC-852-87-R
DF-iPSC-852-94-R

b

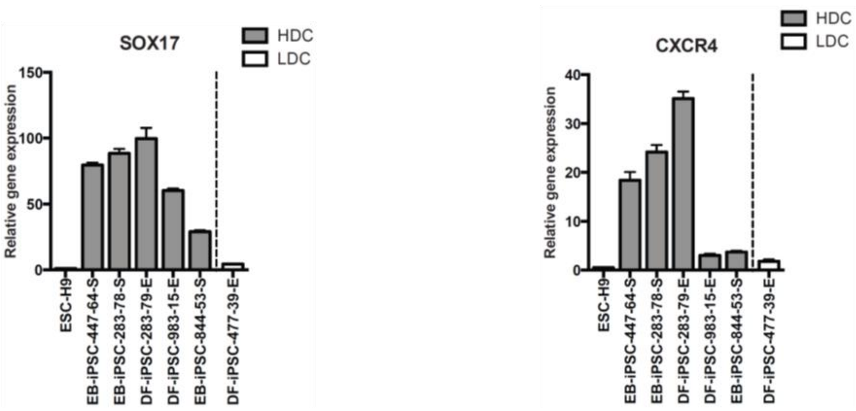
SOX17
CXCR4
HDC
LDC
Relative gene expression
ESC-H9
EB-iPSC-447-64-S
EB-iPSC-283-78-S
DF-iPSC-283-79-E
DF-iPSC-983-15-E
EB-iPSC-844-53-S
DF-iPSC-477-39-E

c

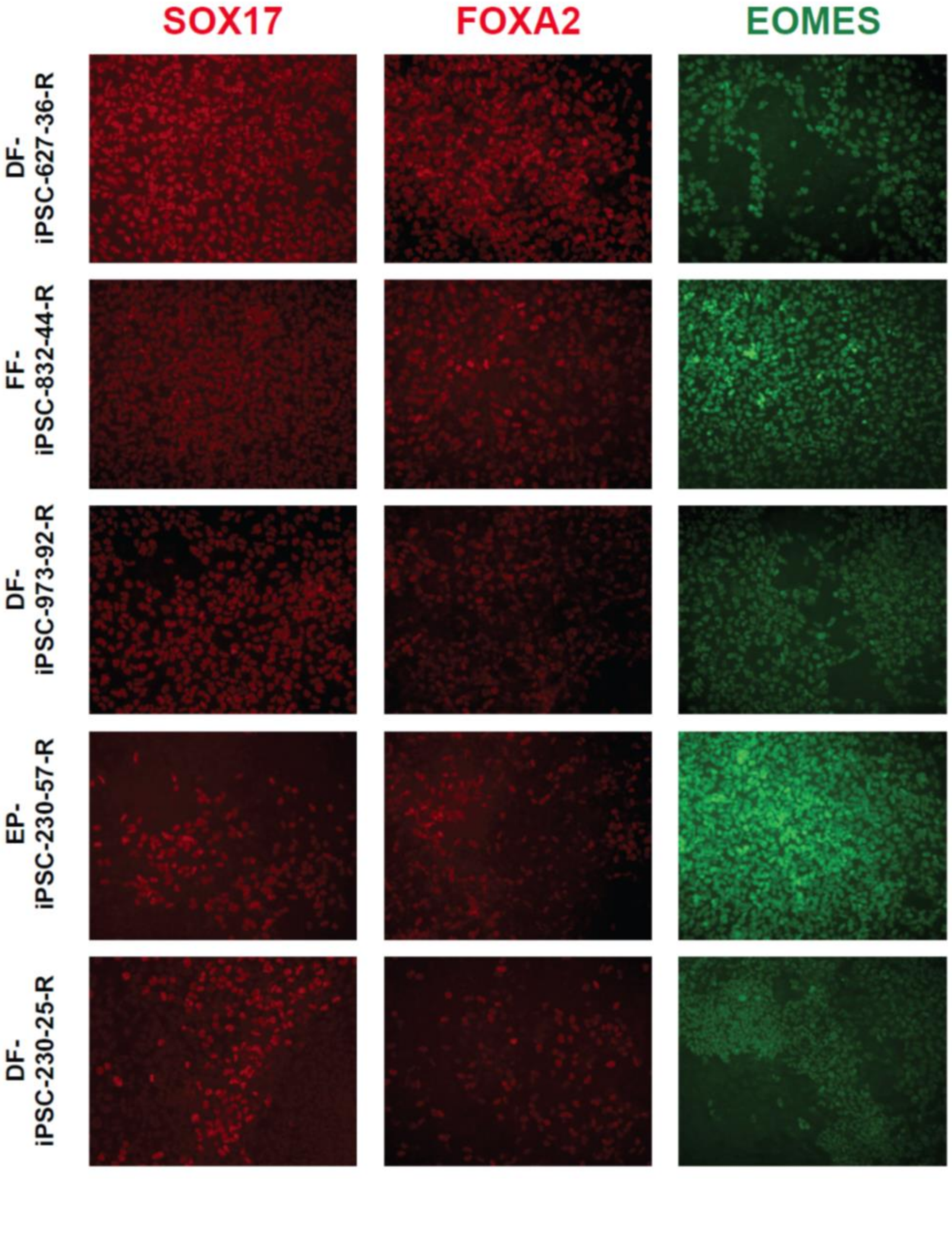
SOX17
FOXA2
EOMES
DF-iPSC-627-36-R
FF-iPSC-832-44-R
DF-iPSC-973-92-R
EP-iPSC-230-57-R
DF-iPSC-230-25-R

d

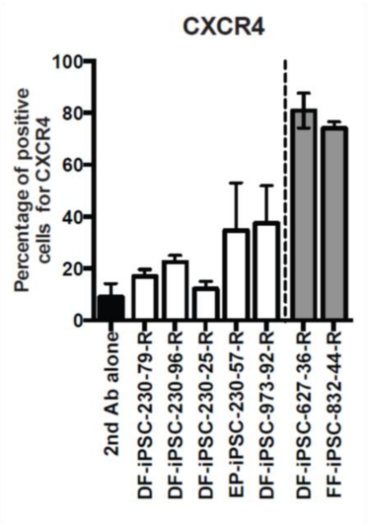

e

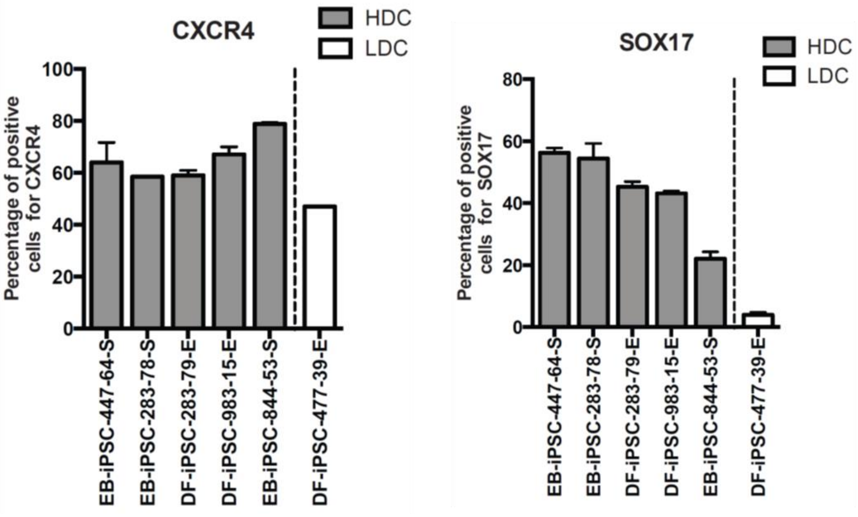

Supplementary Figure 6: Endodermal differentiation in hiPSCs. **(a)** qRT-QPCR analyses show endoderm markers (SOX17 and FOXA2) and primitive streak markers (EOMES and MIXL1) is reduced in LDC hiPSCs in the discovery cohort and hESCs used in the replication chort. **(b)** qRT-PCR analyses show endoderm markers (SOX17 and CXCR4) is reduced in LDC hiPSCs in the replication cohort. **(c)** Reduced immunostaining of SOX17- and FOXA2- positive cells is evident in LDC iPSCs (bottom three rows). **(c)** Flow cytometry for CXCR4 in the discovery cohort and **(d)** CXCR4 and SOX17 in the replication cohort; these figures show that the percentage of cells positive for CXCR4 is less than 50% in LDC iPSCs, while the percentage of SOX17 in the replication cohort is markedly low (<5%) in the LDC iPSCs. Error bars indicate standard deviations from triplicate experiments.

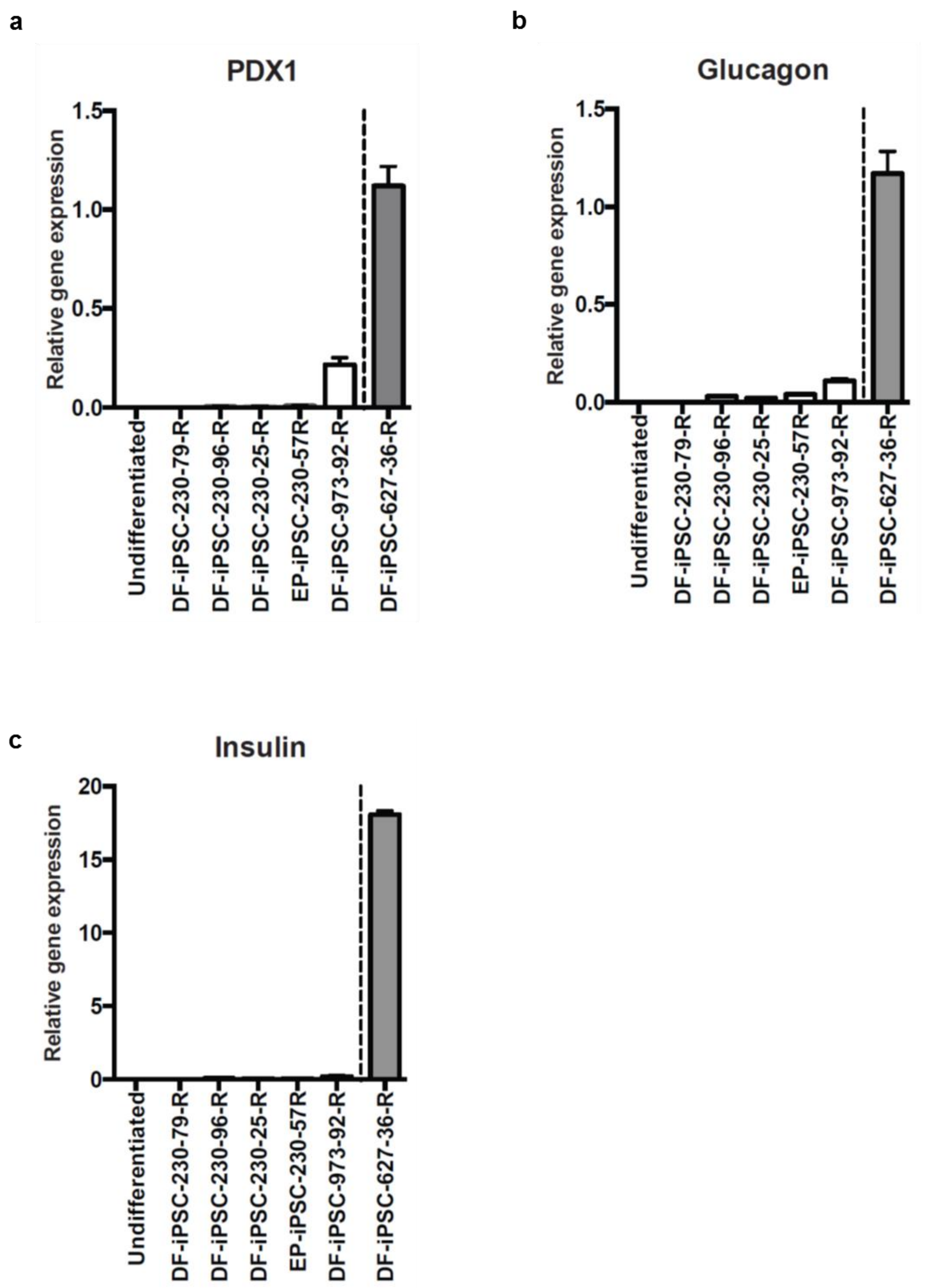

Supplementary Figure 7: Characterisation of hiPSC-derived pancreatic progenitors. **(a)** LDC hiPSC lines were induced to form pancreatic progenitors and qRT-QPCR analysis shows lack of PDX1 expression in these cells. **(b)** After 18 days of differentiation, HDC hiPSC lines produce hormonal cells capable of expressing glucagon and insulin while LDC hiPSC lines do not. Error bars indicate standard deviations from triplicate experiments.

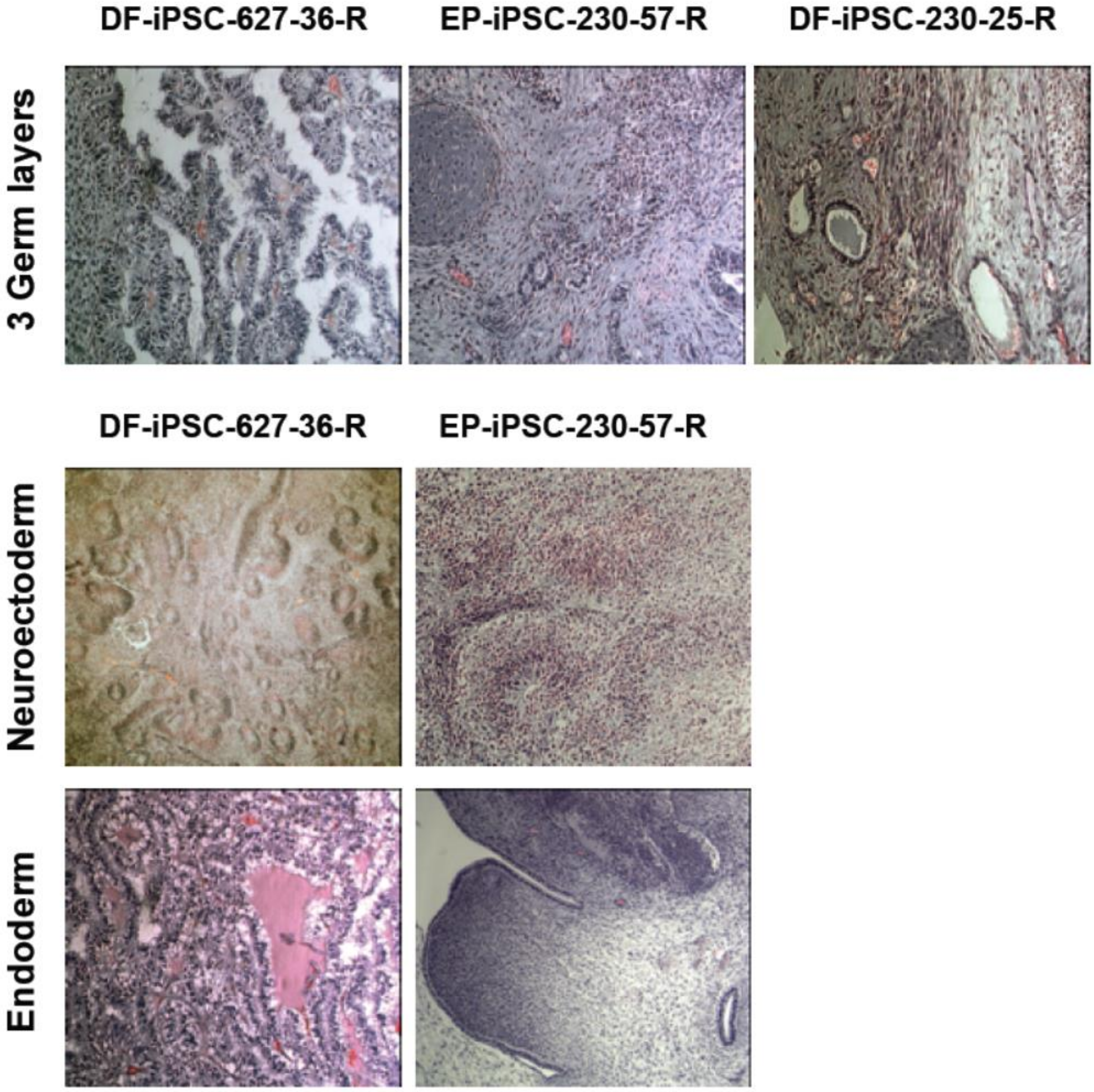

Supplementary Figure 8: Teratoma assays. Teratomas from one HDC- (DF-iPSC-627-36-R) and two LDC- (EP-iPSC-230-57-R & DF-iPSC-230-25-R) hiPSC lines grown on feeders in the presence of knock out serum replacer (KSR) supplemented with FGF2 were injected into the testis capsule of severe combined immunodeficient-beige mice. The tumours were harvested at day 45 after injection. The top row shows the presence of (from left to right) (1) neuroectoderm epithelium (neuroectoderm), (2) cartilage (mesoderm) and pig mental cells (neuroectoderm), and (3) gut epithelium (endoderm). The middle rows show a combination of neuronal rosette and pig mental cells (neuroectoderm). The bottom row shows a homogeneous endoderm epithelium (endoderm).

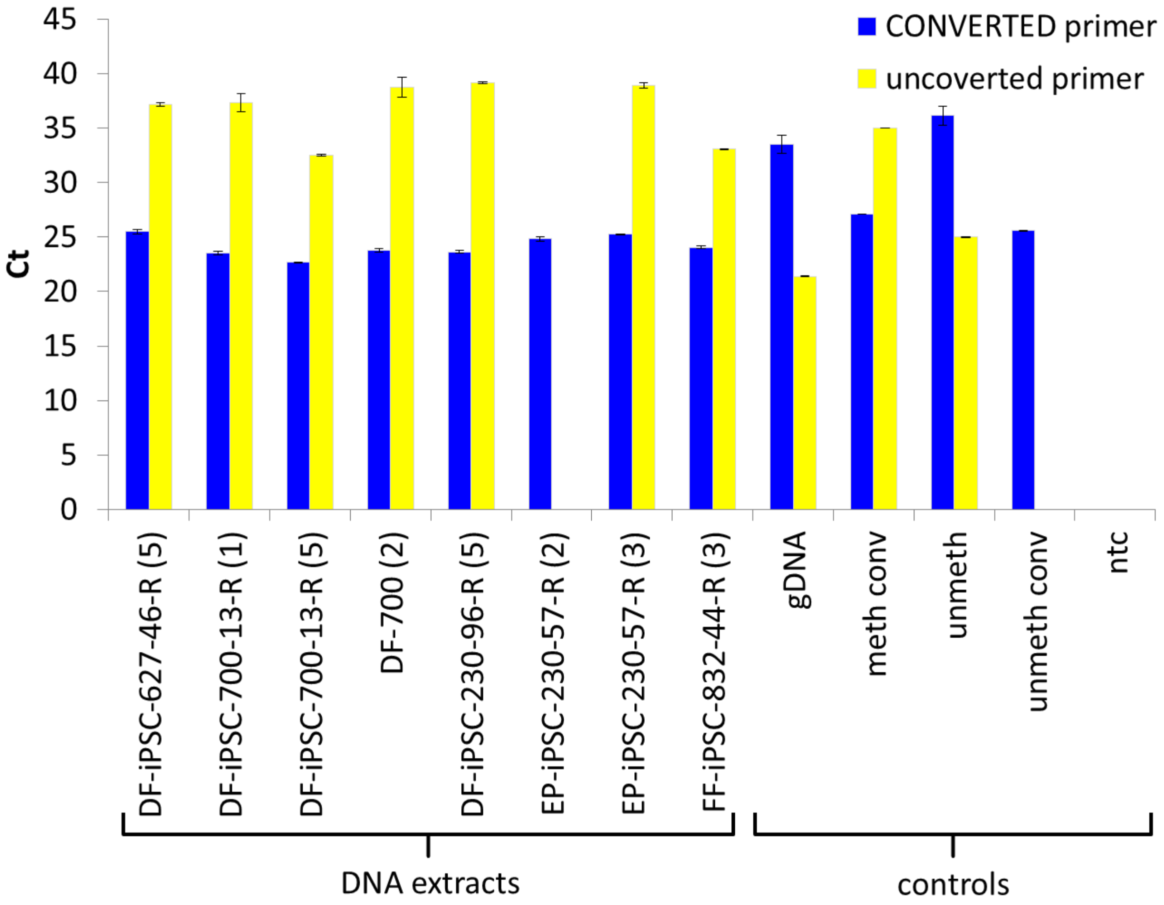

Supplementary Figure 9: Bisulfite conversion analysis by qPCR. Only bisulfite-treated DNA extracts and bisulfite-treated controls ("meth conv" & "unmeth conv") produced amplicons using CONVERTED primers; only untreated genomic DNA ("gDNA") and untreated *in vitro* unmethylated controls ("unmeth") produced amplicons using conventional unconverted primers. Using the difference in Cts between the two primer pairs, we estimated conversion rates of DNA extracts to be greater than 99.8%. Error bars indicate standard errors from triplicate experiments.

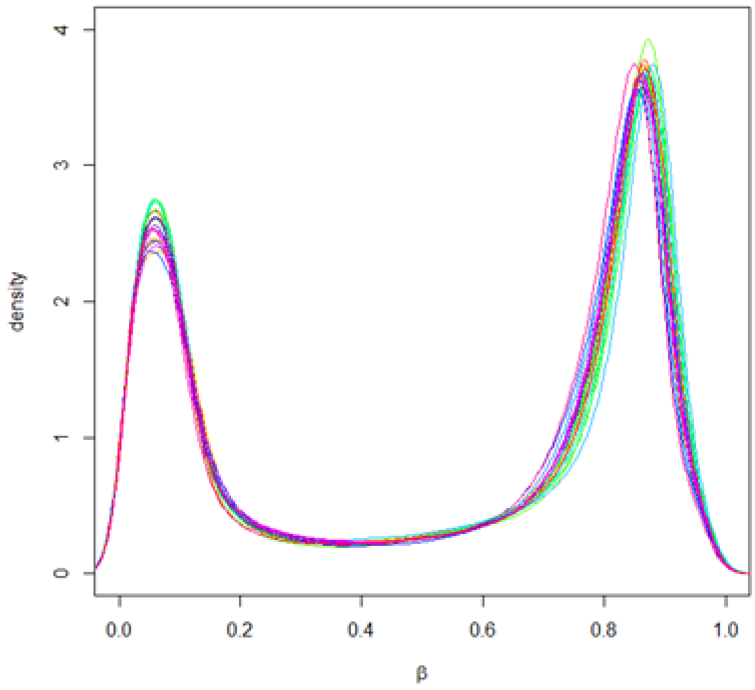

Supplementary Figure 10: Bisulfite conversion analysis by manual inspection of beta plots. The classic bimodal “beta” distribution of CpG methylation levels for the iPSC lines using the Illumina 450K BeadChip, consistent with successful and complete bisulfite conversion.

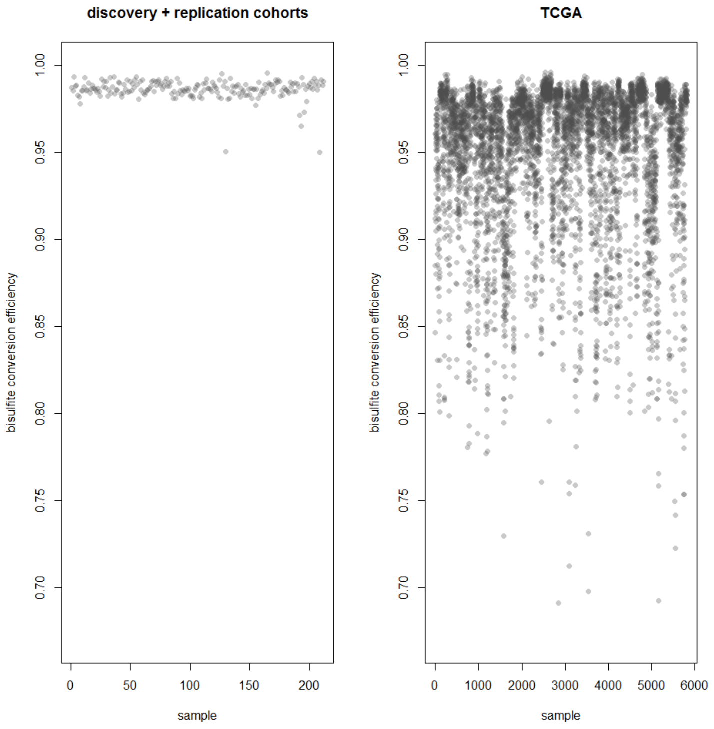

Supplementary Figure 11: Bisulfite conversion analysis using inbuilt control probes on the Illumina 450K BeadChip. Bearing in mind the dynamic range of a microarray is compromised (i.e., the ability to generate β-values close to the extremes of 0 & 1), we estimated conversion to be between 95-100% for all samples. This contrasts favourably with the near-six thousand samples mined from The Cancer Genome Atlas (TCGA)[39] project, for which only 67% of sample produce bisulfite conversion estimates > 95%; however, it is conceivable that many TCGA samples were processed before Illumina's recommendation of a "thermal cycling" protocol, which can reduce estimates of bisulfite conversion with only subtle differences in methylation estimates. Nonetheless, the mean bisulfite conversion estimate in this study (98.6%) was still marginally higher than the TCGA mean bisulfite conversion estimate even after sub-setting the TCGA dataset to include only samples with bisulfite conversion estimates > 95% (97.3%).

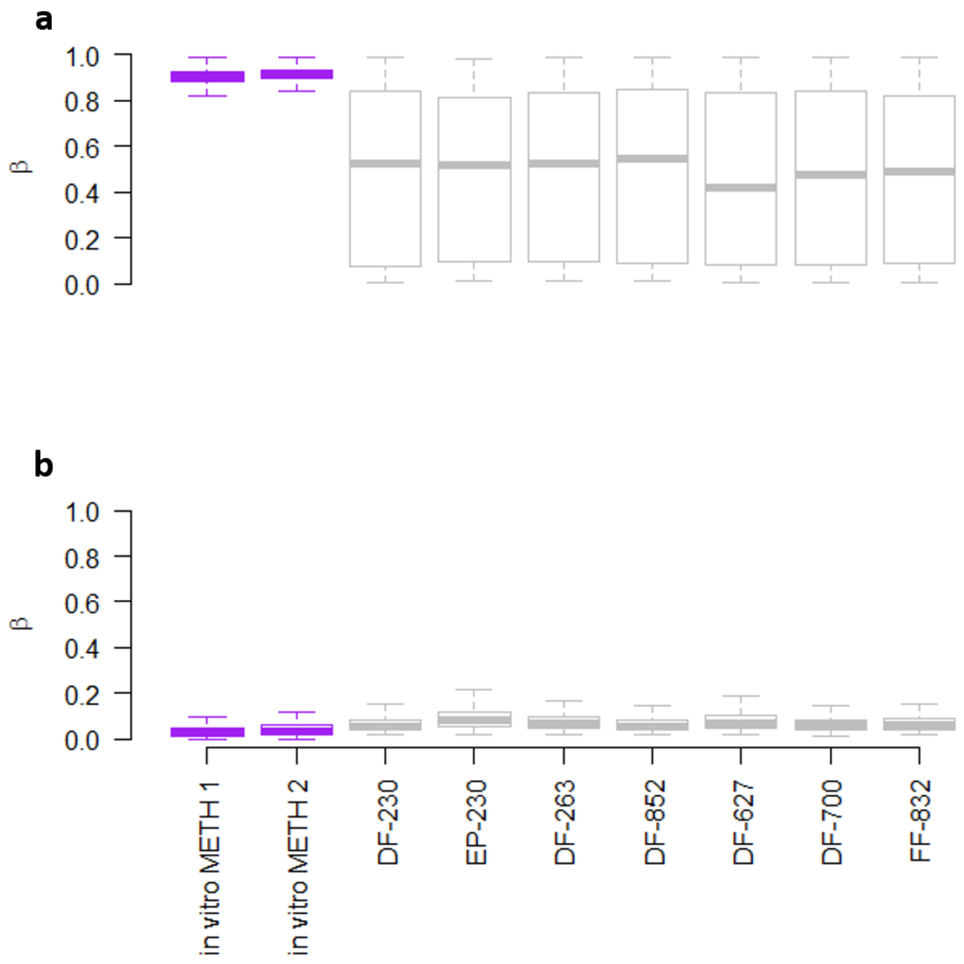

Supplementary Figure 12: Bisulfite conversion analysis using *in vitro* methylated controls. (a) As would be expected under successful bisulfite conversion conditions, *in vitro* methylated control DNA (purple boxplots) produced high β-values for CpG probes (95% of data, $\beta > 0.82$) while differentiated donor cells (grey boxplots) produced a range of β-values. (b) *In vitro* methylated control DNA produced low β-values for non-CG probes (95% of data, $\beta < 0.11$), which is comparable to differentiated donor cells that are known to be unmethylated at non-CG loci. Bisulfite conversion of *in vitro* unmethylated controls was estimated at 97.7% and 98.8% using the inbuilt Illumina 450K BeadChip control probes (cf. Supplementary Figure 5). Because *bone fide* unmethylated non-CG loci produce very low β-values, we can infer that the non-CG methylation signal in the pluripotent stem cells used in this study is real.

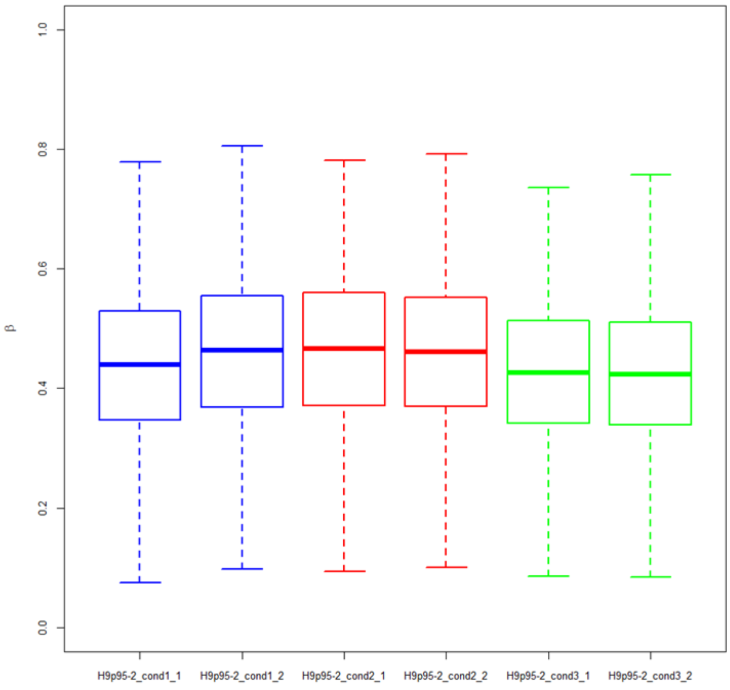

Supplementary Figure 13: Bisulfite conversion analysis by alternative ultra-stringent bisulfite conversion protocols applied to H9 ESC DNA had no effect on non-CG levels. Each of the three protocols were carried out in duplicate.

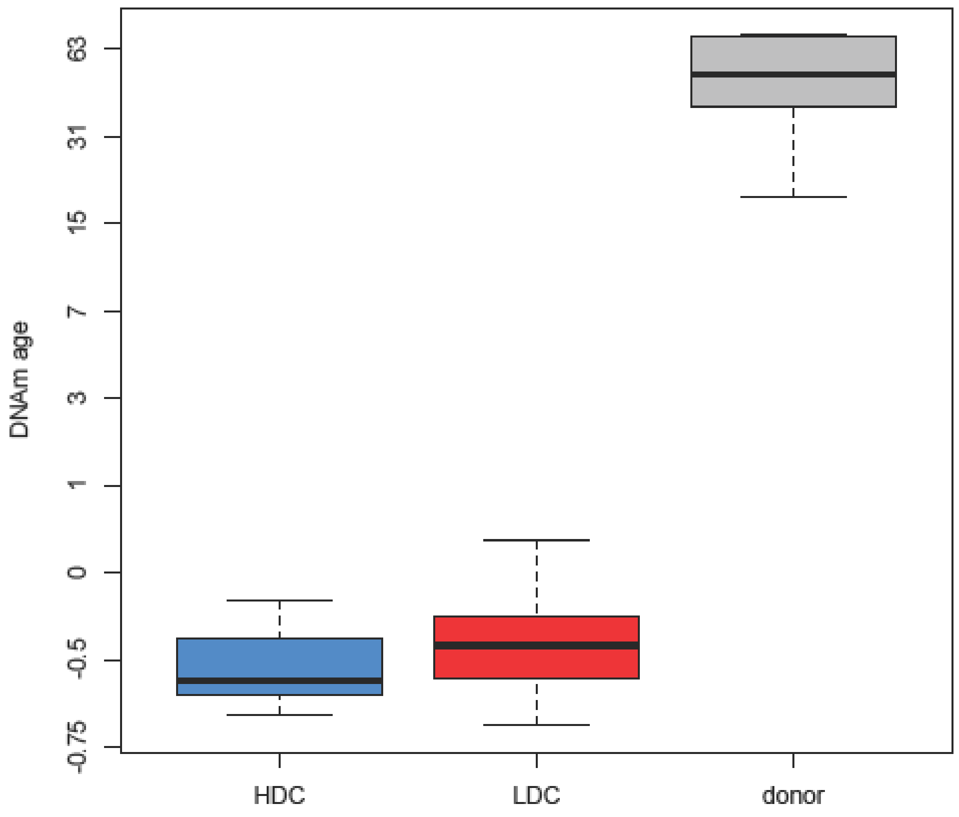

Supplementary Figure 14: Boxplot illustrating DNA methylation age (Horvath, 2013) range of HDC- and LDC- pluripotent stem cells and terminally differentiated donor cell types. Consistent with literature (Horvath, 2013), pluripotent stem cells exhibited negative age indicative of their prenatal origins, while predicted age ranges of donor cell types ranged from 18-70 yrs. There was no significant difference in age between HDC- and LDC- pluripotent stem cells.

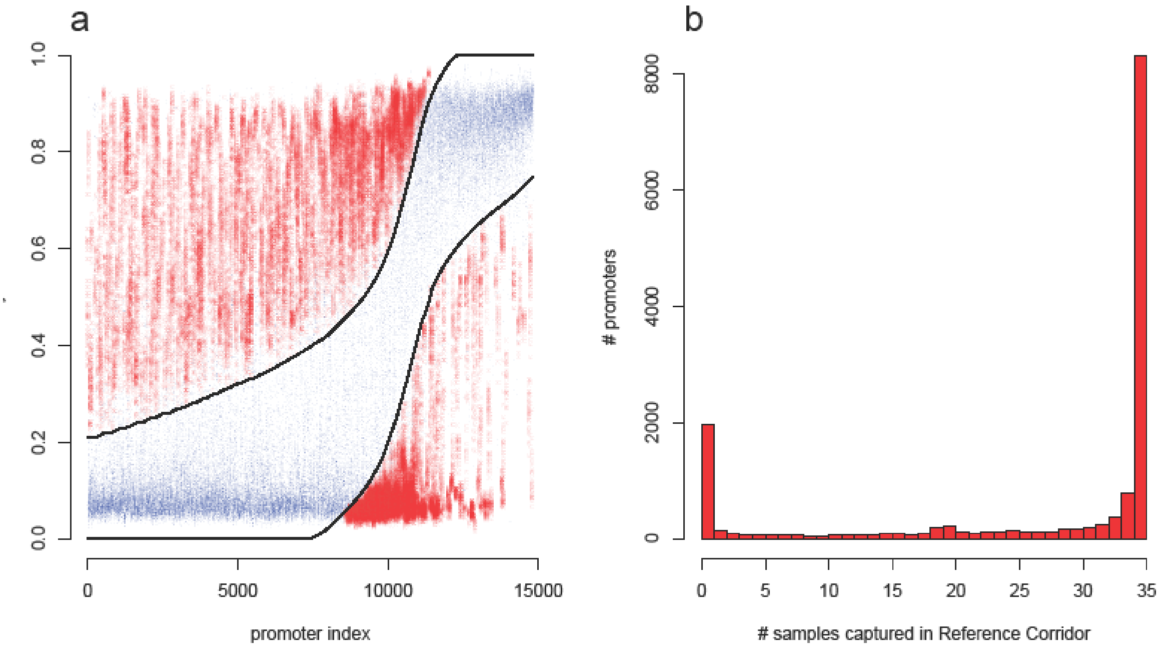

Supplementary Figure 15: Comparison with the 'Reference Corridor' (Bock et al, 2011). The reference corridor refers to methylation thresholds at gene promoters derived from the analysis of 20 ES cell lines using reduced representation bisulfite sequencing. We derived methylation scores by taking the median beta-value of all Illumina 450K BeadChip probes that fell within each of the 14,820 promoters that successfully remapped from NCBI/hg18 to GRCh37/hg19 coordinates. **(a)** Scatterplot illustrating the general agreement between Illumina 450K BeadChip estimates and those of the Reference Corridor. 76.8% of all promoter methylation estimates from the Illumina 450K BeadChip fell within the reference boundaries. Blue dots = promoters capturing at least one methylation estimate from our cohort; red crosshairs = promoters failing to capture any methylation estimate from our cohort. **(b)** Histogram showing the number of cell lines in this study (x-axis) captured by the Reference Corridor thresholds for each promoter tested. The peak at the left of the histogram shows that the Reference Corridors of 1,734 (11.7%) promoters failed to capture any methylation estimate derived from our cohort; the dense patches of red clustering near Reference Corridor boundaries suggests this is largely attributable to the different technologies being compared (Illumina 450K BeadChip vs. RRBS).

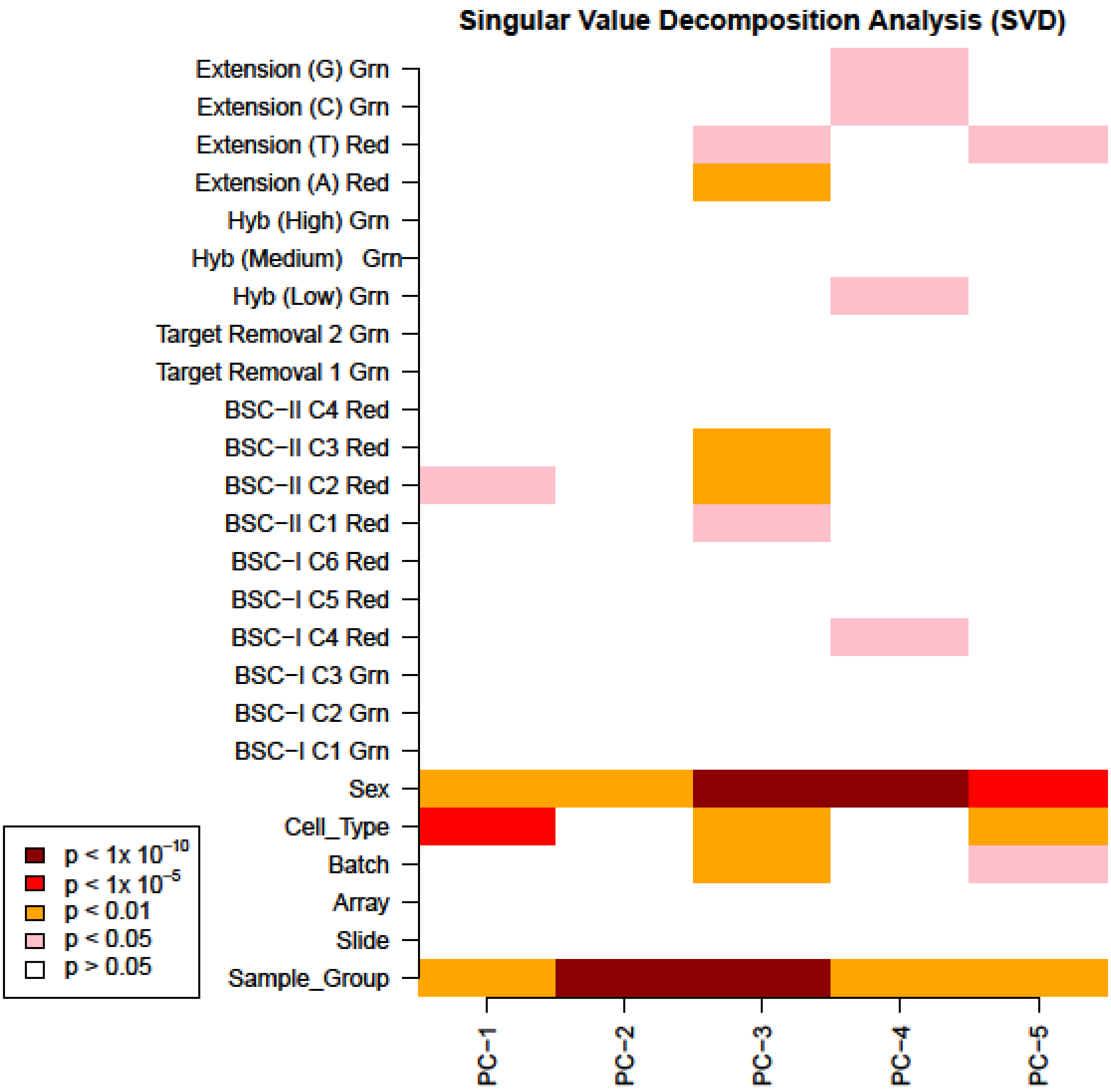

Supplementary Figure 16: Singular Value Decomposition (SVD) analysis to identify significant principal components of variation (x-axis) due to confounding factors (y-axis) in the discovery cohort. Three factors reflect biological variation: "Sex" (male or female), "Cell_Type" (pluripotent or donor), and "Sample_Group" (1 of 32 cell lines [see **Table S1**]). The remaining factors reflect technical variation: "Array" (array position), "Slide" (a collection of arrays), "Batch" (slides processed together) and the first 19 variables (control probes on the Illumina 450K BeadChip).

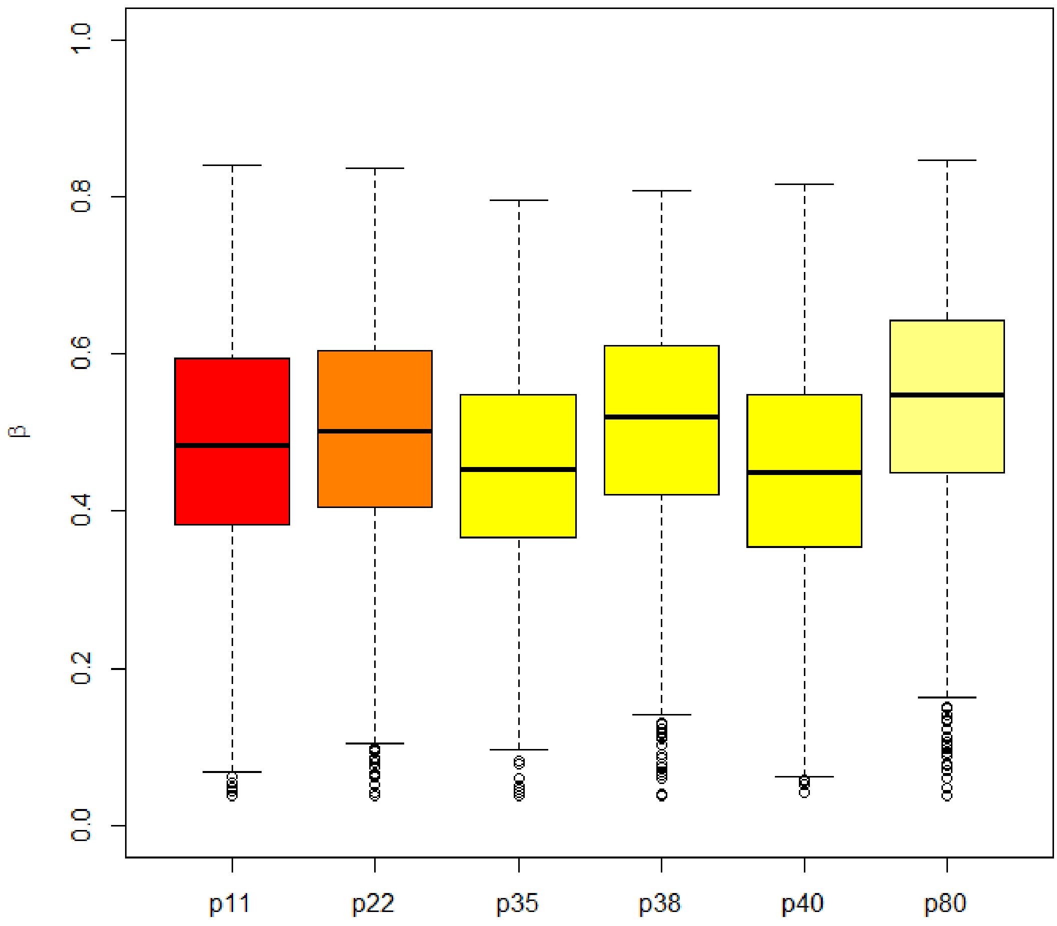

Supplementary Figure 17: Non-CG methylation in iPSC lines is not associated with passage number. All iPSC lines were selected from the HDC group to avoid confounding.

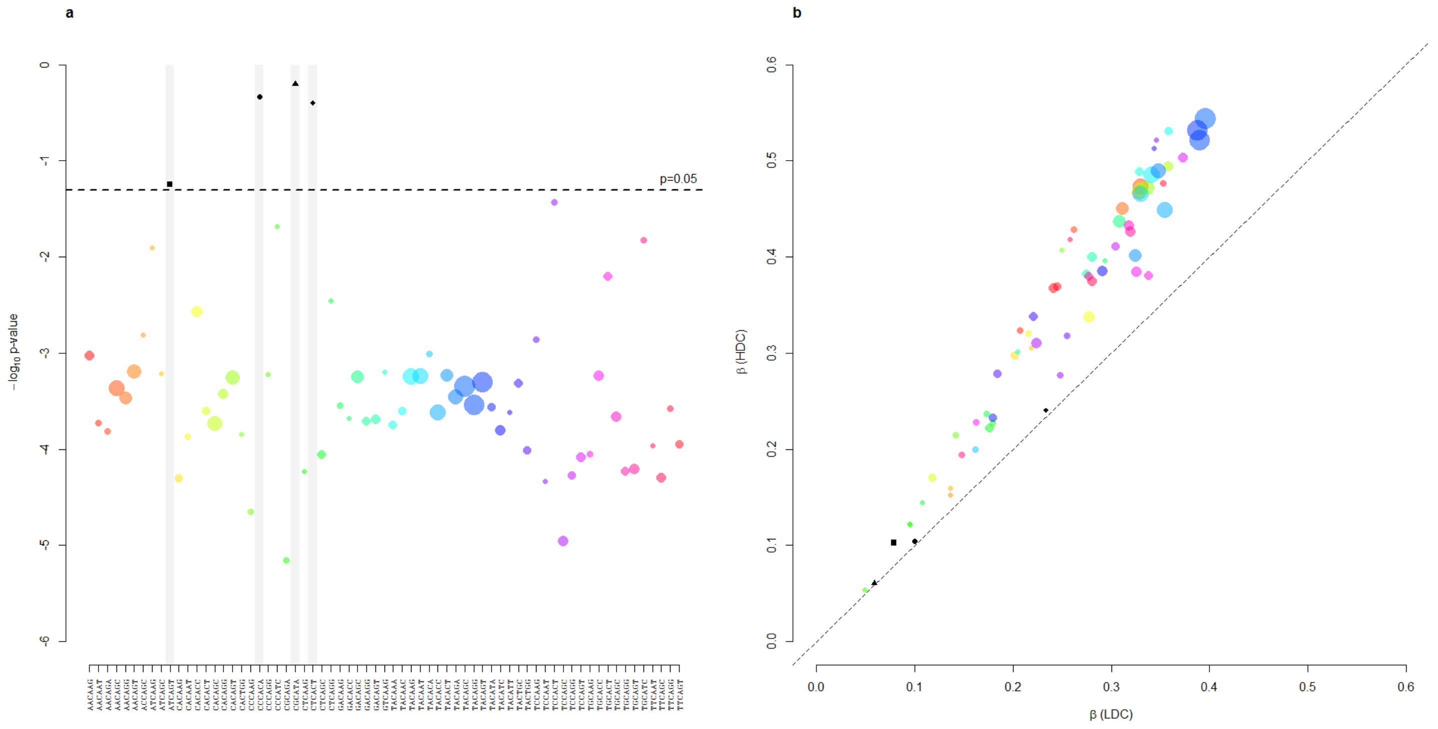

Supplementary Figure 18: Non-CG methylation as a biomarker for differentiation capacity in iPSCs is not motif-specific. **(a)** Differences in mean non-CG methylation levels between LDC- and HDC- iPSCs were significant for virtually all (94%) non-CG motifs (non-significant motifs highlighted). **(b)** Mean β-values for LDC- (x-axis) and HDC- (y-axis) iPSCs plotted for each motif. The colours of dots are conserved between figures; the sizes of dots are size-scaled ($\log_{10}$) according to the number of motifs analysed (N=1 – 830).

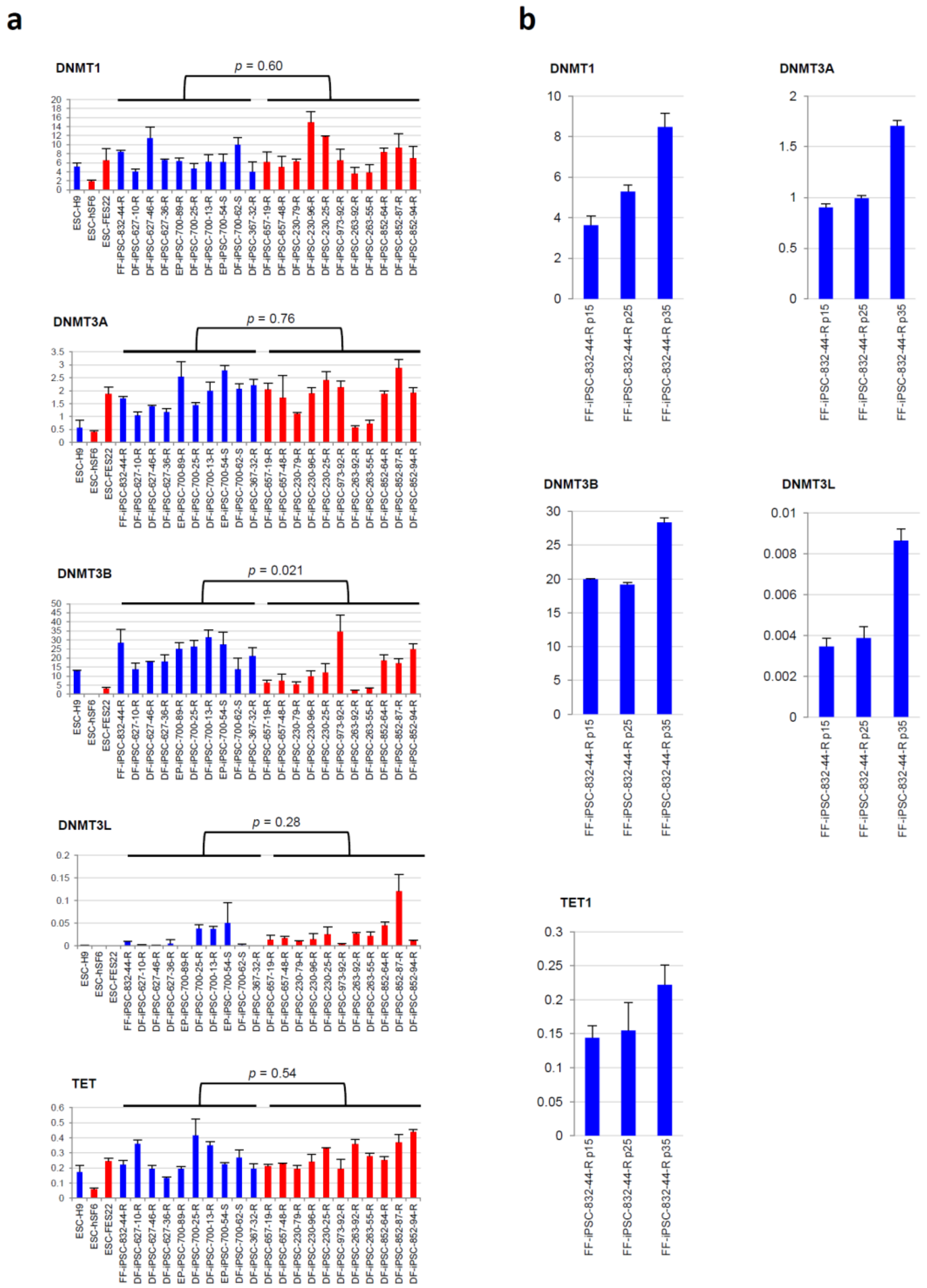

Supplementary Figure 19: Gene expression profiling for the *DNMT* genes and *TET1* by RT-PCR. **(a)** Gene expression for each individual cell line in the discovery cohort. Error bars indicate standard deviation using technical triplicates. *DNMT3B* gene expression was significantly lower in the LDC group compared with the HDC group. **(b)** Effect of passage number on DNMT genes and TET1 by RT-PCR. Error bars indicate standard deviations from triplicate experiments.

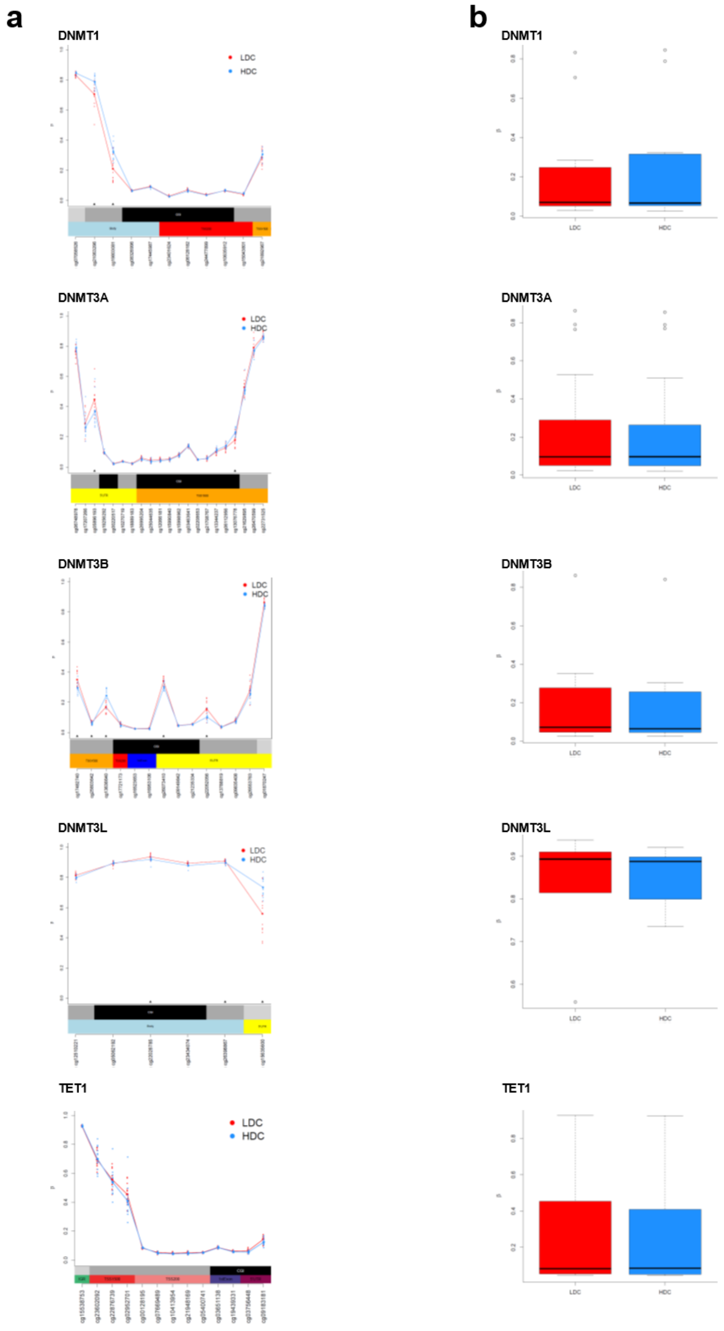

Supplementary Figure 20: **(a)** CpG methylation scores of probes tiled across the promoter regions (-5 kb to +1 kb) of *DNMT1*, *DNMT3A*, *DNMT3B*, *DNMT3L* and *TET1* on the Illumina 450K BeadChip for LDC- and HDC- iPSC lines used in the discovery cohort. Significantly differentially methylated probes are marked as black triangles. **(b)** Boxplots of aggregated CpG methylation scores across promoters for the cell lines used in the discovery cohort. The differences in mean methylation between LDC- and HDC- iPSCs across the promoter regions were not significant.

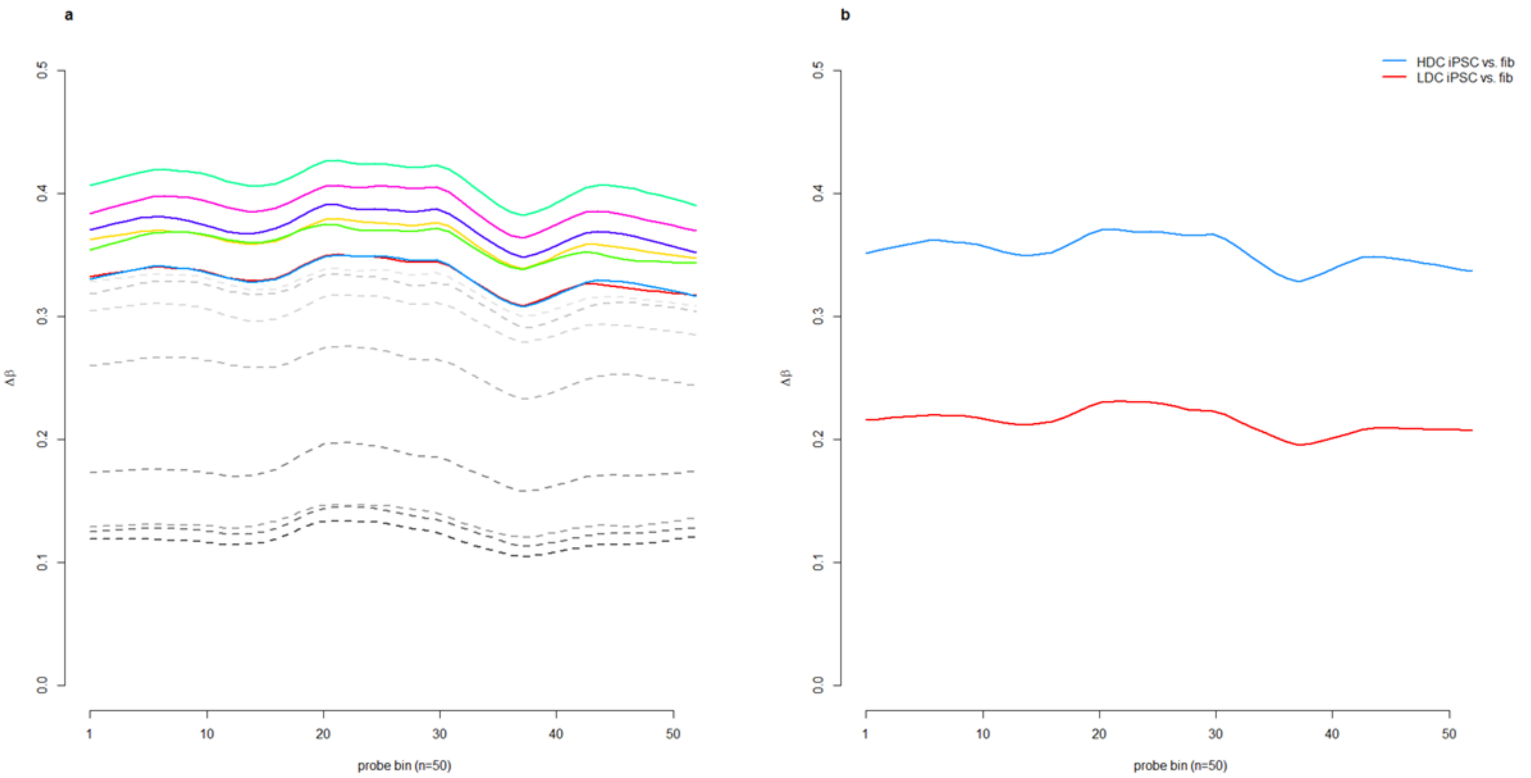

Supplementary Figure 21: HDC iPSC lines show a greater magnitude of *de novo* methylation at non-CG sites after reprogramming compared with LDC iPSC lines. **(a)** Δβ between iPSC and matched donor cell lines. Δβ-values for HDC iPSC lines are solid, colored lines; Δβ-values for LDC iPSC lines are dashed, grey-scale lines. **(b)** As (**a**) but summarised across LDC- and HDC- iPSC groups for clarity. Loess-smoothed curves have been fitted to the data in each figure. Each data point represents $\Delta\beta_{mean}$ for a bin of 50 randomly sampled (without replacement) non-CG probes; aligned bins span the full dataset of non-CG probes.

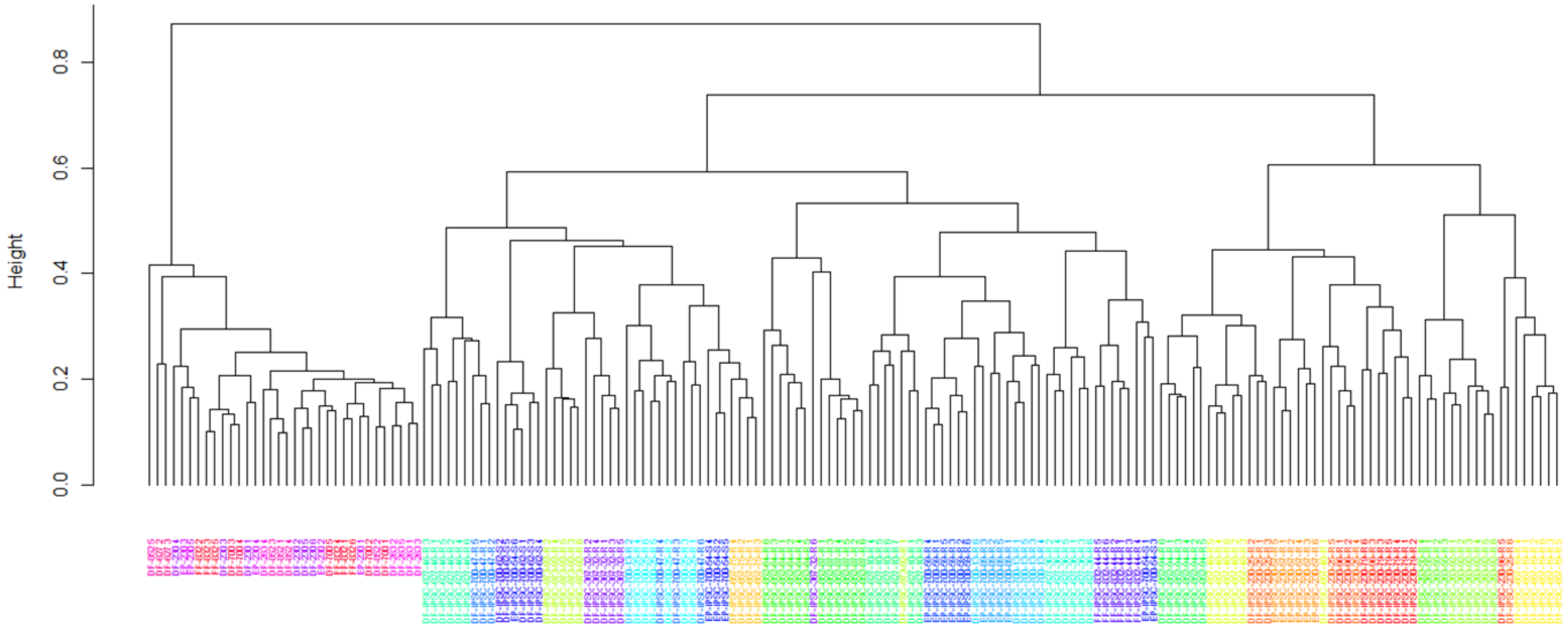

Supplementary Figure 22: Dendrogram illustrating tight clustering of technical replicates (technical replicates are unified by color).

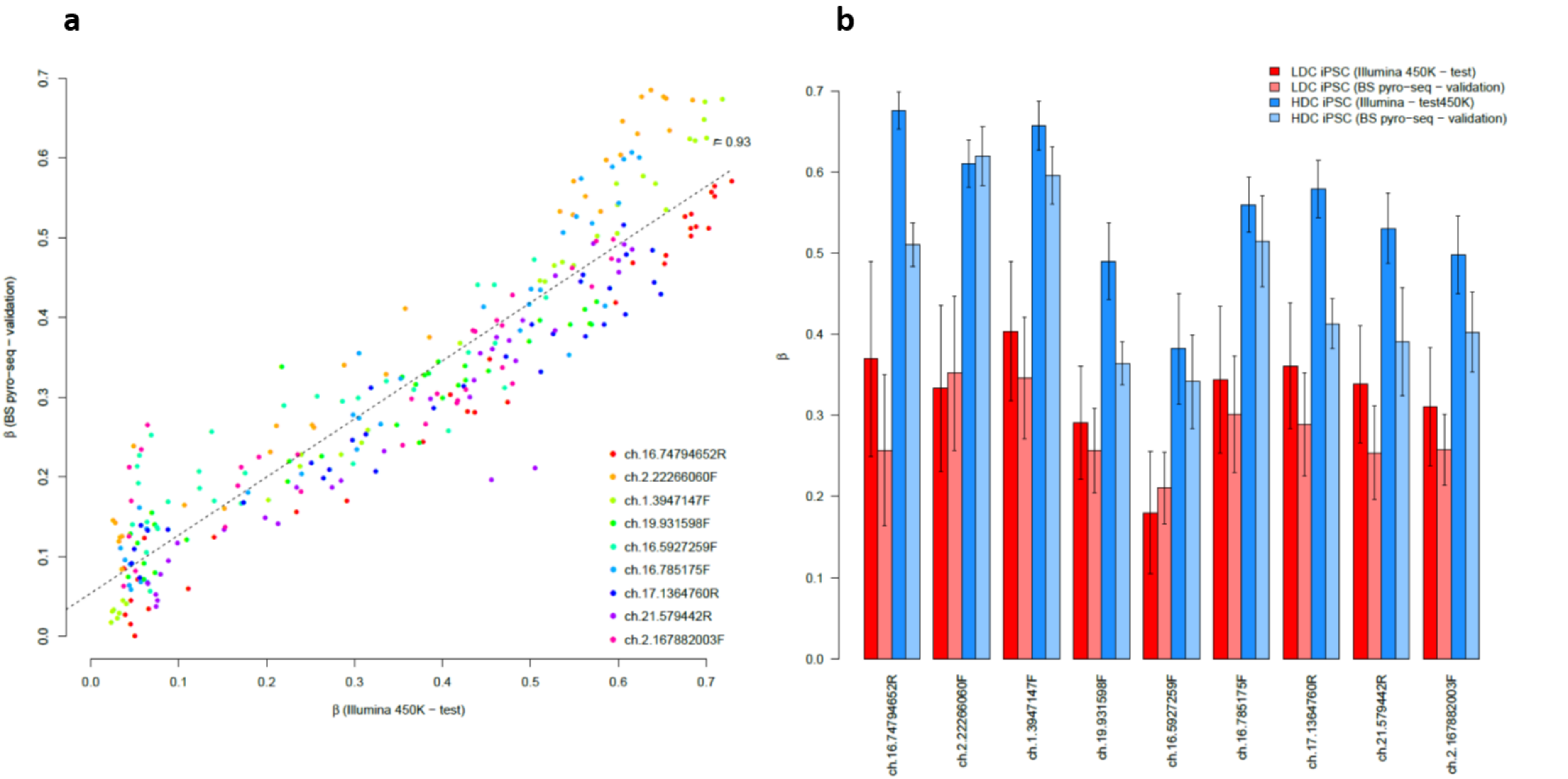
a
b
r= 0.93
ch.16.74794652R
ch.2.22266060F
ch.1.3947147F
ch.19.931598F
ch.16.5927259F
ch.16.785175F
ch.17.1364760R
ch.21.579442R
ch.2.167882003F
β (Illumina 450K – test)
β (BS pyro–seq – validation)
LDC iPSC (Illumina 450K – test)
LDC iPSC (BS pyro–seq – validation)
HDC iPSC (Illumina – test450K)
HDC iPSC (BS pyro–seq – validation)
β

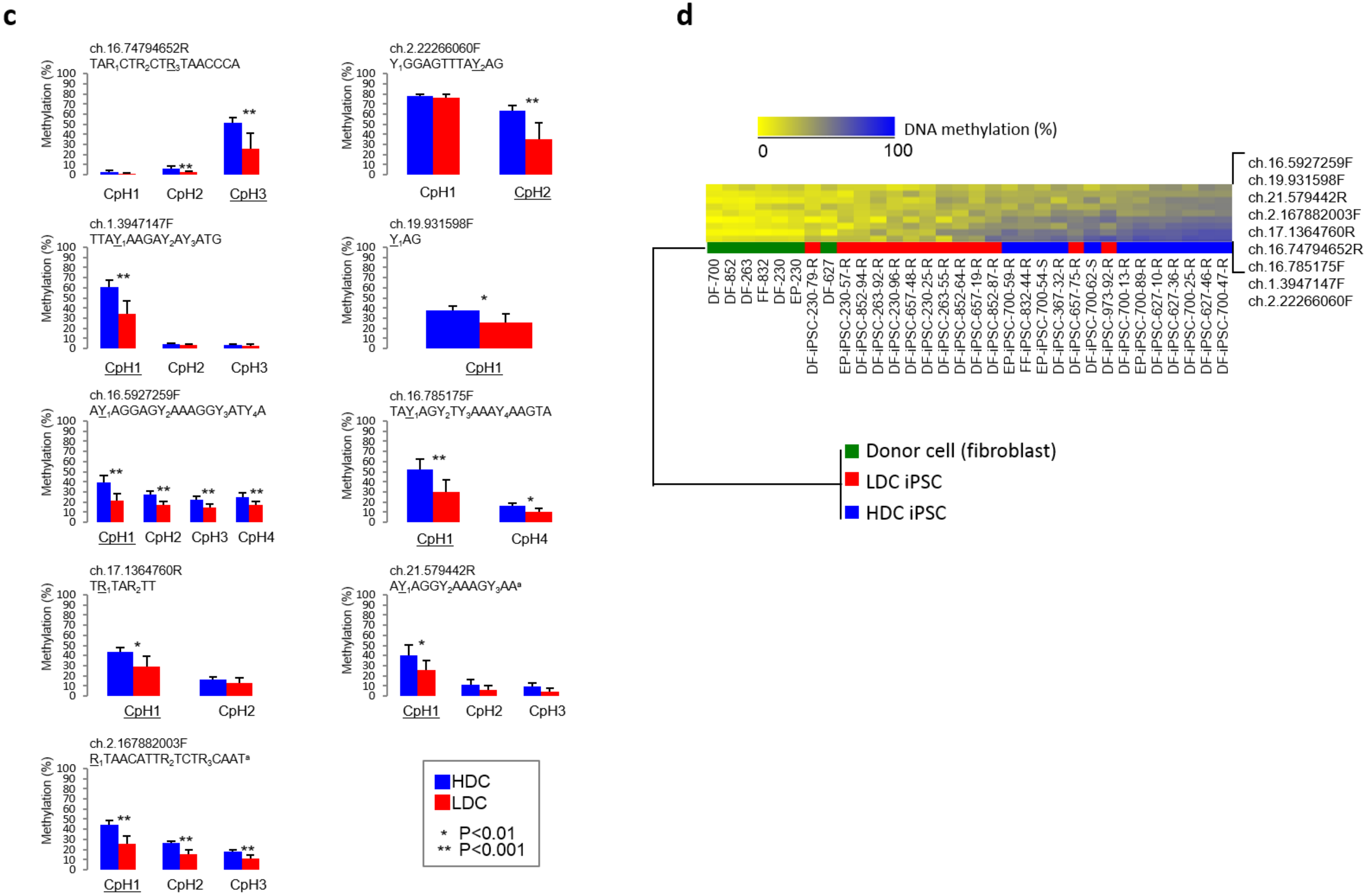
c
ch.16.74794652R
TAR$_1$CTR$_2$CTR$_3$TAACCCA
Methylation (%)
CpH1
CpH2
CpH3
ch.2.22266060F
Y$_1$GGAGTTTAY$_2$AG
CpH1
CpH2
ch.1.3947147F
TTAY$_1$AAGAY$_2$AY$_3$ATG
CpH1
CpH2
CpH3
ch.19.931598F
Y$_1$AG
CpH1
ch.16.5927259F
AY$_1$AGGAGY$_2$AAAGGY$_3$ATY$_4$A
CpH1
CpH2
CpH3
CpH4
ch.16.785175F
TAY$_1$AGY$_2$TY$_3$AAAY$_4$AAGTA
CpH1
CpH4
ch.17.1364760R
TR$_1$TAR$_2$TT
CpH1
CpH2
ch.21.579442R
AY$_1$AGGY$_2$AAAGY$_3$AA[a]
CpH1
CpH2
CpH3
ch.2.167882003F
R$_1$TAACATTR$_2$TCTR$_3$CAAT[a]
CpH1
CpH2
CpH3
HDC
LDC
* P<0.01
** P<0.001
d
DNA methylation (%)
0
100
ch.16.5927259F
ch.19.931598F
ch.21.579442R
ch.2.167882003F
ch.17.1364760R
ch.16.74794652R
ch.16.785175F
ch.1.3947147F
ch.2.22266060F
DF-700
DF-852
DF-263
FF-832
DF-230
EP-230
DF-iPSC-230-79-R
DF-627
EP-iPSC-230-57-R
DF-iPSC-852-94-R
DF-iPSC-263-92-R
DF-iPSC-230-96-R
DF-iPSC-657-48-R
DF-iPSC-230-25-R
DF-iPSC-263-55-R
DF-iPSC-852-64-R
DF-iPSC-657-19-R
DF-iPSC-852-87-R
EP-iPSC-700-59-R
FF-iPSC-832-44-R
EP-iPSC-700-54-S
DF-iPSC-367-32-R
DF-iPSC-657-75-R
DF-iPSC-700-62-S
DF-iPSC-973-92-R
DF-iPSC-700-13-R
EP-iPSC-700-89-R
DF-iPSC-627-10-R
DF-iPSC-627-36-R
DF-iPSC-700-25-R
DF-iPSC-627-46-R
DF-iPSC-700-47-R
Donor cell (fibroblast)
LDC iPSC
HDC iPSC

Supplementary Figure 23: Validation of the nine-probe non-CG differentiation capacity signature. **(a)** Scatter plot illustrating the agreement for DNA methylation estimates between two independent technologies (Illumina 450K BeadChips and bisulfite pyrosequencing). **(b)** DNA methylation levels for each MVP of the nine-probe signature in LDC- and HDC- iPSCs using Illumina 450K BeadChips (red and blue bars) and bisulfite pyrosequencing (pale-red and pale-blue bars). Error bars are 95% confidence intervals. **(c)** DNA methylation levels for non-CG sites covered by each pyrosequencing assay. Assays were designed to target MVPs discovered using Illumina 450K BeadChips (underlined on the x-axis). Sequence context for each MVP is provided above each plot. Four of the nine assays revealed additional, novel, and significant non-CG loci. Error bars indicate standard errors from triplicate experiments. **(d)** Heatmap illustrating the methylation levels ascertained by bisulfite pyrosequencing of each non-CG site in the nine-probe signature for iPSCs and donor cells, ordered by average methylation level.

cohort	donor ID	donor cell type	reprogramming method	sex	# colony replicates (post QC)	differentiation capacity	cell line type	cell line name	cell line ID
Donor	COX	DF	NA	F	6	NA	Fibroblast	COXS Fibroblast	DF-230
Donor	COX	EP	NA	F	5	NA	Fibroblast	COXV Fibroblast	EP-230
Donor	TWa	DF	NA	F	5	NA	Fibroblast	TWa Fibroblast	DF-263
Donor	TWb	DF	NA	F	5	NA	Fibroblast	TWb Fibroblast	DF-852
Donor	BBHX	DF	NA	F	2	NA	Fibroblast	BBHX Fibroblast	DF-627
Donor	A1ATD	DF	NA	M	5	NA	Fibroblast	A1ATD Fibroblast	DF-700
Donor	hIPSC40	FF	NA	M	6	NA	Fibroblast	hIPSC40 Fibroblast	FF-832
Discovery	COX	DF	R	F	6	LDC	iPSC	COXS5	DF-iPSC-230-79-R
Discovery	COX	DF	R	F	4	LDC	iPSC	COXS6	DF-iPSC-230-96-R
Discovery	COX	DF	R	F	6	LDC	iPSC	COXS8	DF-iPSC-230-25-R
Discovery	COX	EP	R	F	6	LDC	iPSC	COXV3	EP-iPSC-230-57-R
Discovery	TM	DF	R	M	4	LDC	iPSC	NTM	DF-iPSC-657-75-R
Discovery	TM	DF	R	M	6	LDC	iPSC	OTM	DF-iPSC-657-19-R
Discovery	TM	DF	R	M	6	LDC	iPSC	OTM6	DF-iPSC-657-48-R
Discovery	4C1	DF	R	F	6	LDC	iPSC	4C1	DF-iPSC-973-92-R
Discovery	TWa	DF	R	F	6	LDC	iPSC	TWa1	DF-iPSC-263-92-R

Discovery	TWa	DF	R	F	4	LDC	iPSC	TWa3	DF-iPSC-263-55-R
Discovery	TWb	DF	R	F	6	LDC	iPSC	TWb1	DF-iPSC-852-64-R
Discovery	TWb	DF	R	F	6	LDC	iPSC	TWb2	DF-iPSC-852-87-R
Discovery	TWb	DF	R	F	6	LDC	iPSC	TWb3	DF-iPSC-852-94-R
Discovery	BBHX	DF	R	F	6	HDC	iPSC	BBHX5	DF-iPSC-627-10-R
Discovery	BBHX	DF	R	F	6	HDC	iPSC	BBHX6	DF-iPSC-627-46-R
Discovery	BBHX	DF	R	F	6	HDC	iPSC	BBHX8	DF-iPSC-627-36-R
Discovery	A1ATD	DF	R	M	6	HDC	iPSC	A1ATD7B RT Corrected	DF-iPSC-700-25-R
Discovery	A1ATD	DF	R	M	6	HDC	iPSC	A1ATDC2 RT Corrected	DF-iPSC-700-13-R
Discovery	A1ATD	EP	R	M	4	HDC	iPSC	A1ATD7 EP	EP-iPSC-700-89-R
Discovery	A1ATD	DF	R	M	6	HDC	iPSC	A1ATD7 LP	DF-iPSC-700-47-R
Discovery	A1ATD	EPC	R	M	6	HDC	iPSC	A1ATD EPC	EP-iPSC-700-59-R
Discovery	A1ATD	EPC	S	M	6	HDC	iPSC	A1ATD SV EPC	EP-iPSC-700-54-S
Discovery	A1ATD	DF	S	M	5	HDC	iPSC	A1ATD SV Fibroblast	DF-iPSC-700-62-S
Discovery	hIPSC40	FF	R	M	6	HDC	iPSC	hIPSC40	FF-iPSC-832-44-R
Discovery	7C10	DF	R	M	6	HDC	iPSC	7C10	DF-iPSC-367-32-R
Replication	FES22	ESC	NA	M	3	LDC	ESC	FES22	ESC-FES22
Replication	hSF6	ESC	NA	F	5	LDC	ESC	hSF6	ESC-hSF6
Replication	H9p95	ESC	NA	F	5	HDC	ESC	H9p95	ESC-H9

Replication	FS13	DF	E	M	1	LDC	iPSC	FSFE13.C	DF-iPSC-447-39-E
Replication	FS12	EB	S	F	1	LDC	iPSC	FSPS12.A	EB-iPSC-685-68-S
Replication	FS18	DF	E	M	1	HDC	iPSC	FSFE18.C	DF-iPSC-283-79-E
Replication	FS10	DF	E	F	1	HDC	iPSC	FSFE10.A	DF-iPSC-983-15-E
Replication	FS13	EB	S	M	1	HDC	iPSC	FSPS13.B	EB-iPSC-447-64-S
Replication	FS18	EB	S	M	1	HDC	iPSC	FSPS18.B	EB-iPSC-283-78-S
Replication	FS11	EB	S	M	1	HDC	iPSC	FSPS11.B	EB-iPSC-844-53-S

Supplementary Table 1: Summary of the cell lines assayed on Illumina 450K BeadChips. A total of 197 arrays were run; 2 arrays failed QC. Where appropriate, methylation levels (β) for each cell line ("sample") were calculated as the average across plate replicates ("DNA extracts"). Key: DF = dermal fibroblast, EP = endothelial precursor, EPC = endothelial progenitor cell, FF = foreskin fibroblast, EB = erythroblast, ESC = embryonic stem cell; R = retroviral, S = Sendai virus, E = episomal; F = female, M = male.

MVP	Illumina Infinium 450K Human Methylation BeadChip				Bisulfite Pyrosequencing			
	**β LDC (95% CI)**	**β HDC (95% CI)**	**Δβ (95%CI)**	***p*-value**	**β LDC (95% CI)**	**β HDC (95% CI)**	**Δβ (95%CI)**	***p*-value**
ch.16.74794652R	0.37 (0.26-0.48)	0.67 (0.63-0.71)	0.30 (0.19-0.42)	$5.3x10^{-5}$	0.26 (0.16-0.35)	0.53 (0.51-0.56)	0.28 (0.18-0.37)	$2.6x10^{-5}$
ch.2.22266060F	0.34 (0.25-0.43)	0.61 (0.56-0.66)	0.27 (0.17-0.37)	$2.6x10^{-5}$	0.35 (0.26-0.45)	0.66 (0.63-0.68)	0.30 (0.21-0.40)	$1.1x10^{-5}$
ch.1.3947147F	0.40 (0.33-0.47)	0.66 (0.61-0.71)	0.26 (0.18-0.34)	$3.4x10^{-6}$	0.35 (0.27-0.42)	0.63 (0.60-0.66)	0.29 (0.21-0.37)	$1.1x10^{-6}$
ch.19.931598F	0.29 (0.23-0.35)	0.52 (0.44-0.59)	0.23 (0.14-0.32)	$4.7x10^{-5}$	0.26 (0.20-0.31)	0.40 (0.38-0.41)	0.14 (0.09-0.19)	$7.0x10^{-5}$
ch.16.5927259F	0.19 (0.12-0.25)	0.42 (0.34-0.51)	0.24 (0.14-0.33)	$1.2x10^{-4}$	0.21 (0.17-0.25)	0.39 (0.32-0.46)	0.18 (0.11-0.25)	$1.5x10^{-4}$
ch.16.785175F	0.34 (0.26-0.42)	0.57 (0.50-0.63)	0.23 (0.13-0.32)	$8.1x10^{-5}$	0.30 (0.23-0.37)	0.58 (0.55-0.61)	0.28 (0.20-0.35)	$1.1x10^{-6}$
ch.17.1364760R	0.36 (0.29-0.42)	0.59 (0.52-0.65)	0.23 (0.14-0.32)	$2.7x10^{-5}$	0.29 (0.23-0.35)	0.43 (0.40-0.47)	0.14 (0.08-0.21)	$3.9x10^{-4}$
ch.21.579442R	0.33 (0.27-0.40)	0.54 (0.47-0.61)	0.20 (0.12-0.29)	$1.4x10^{-4}$	0.25 (0.20-0.31)	0.45 (0.40-0.50)	0.19 (0.12-0.27)	$2.1x10^{-5}$
ch.2.167882003F	0.31 (0.25-0.37)	0.52 (0.45-0.59)	0.21 (0.13-0.30)	$7.8x10^{-5}$	0.26 (0.21-0.30)	0.45 (0.42-0.49)	0.20 (0.15-0.25)	$2.7x10^{-7}$
MVP aggregate	0.32 (0.26-0.37)	0.57 (0.51-0.62)	0.24 (0.16-0.32)	$4.2x10^{-6}$	0.28 (0.23-0.34)	0.48 (0.44-0.52)	0.20 (0.14-0.26)	$2.7x10^{-6}$

Supplementary Table 2: Confirmation of significant association for nine MVPs that distinguish HDC iPSCs from LDC iPSCs. The Illumina platform was used in the initial phase, while bisulfite pyrosequencing was used as an independent analysis platform for validation.

target gene	primer	primer sequence 5´ to 3´
RPII	F<br>R	GCACCACGTCCAATGACAT<br>GTGCGGTGCTTCCATAA
PBGD	F<br>R	GGAGCCATGTCTGGTAACGG<br>CCACGCGAATCACTCTCATCT
OCT4	F<br>R	AGTGAGAGGCAACCTGGAGA<br>ACACTCGGACCACATCCTTC
NANOG	F<br>R	CATGAGTGTGGATCCAGCTTG<br>CCTGAATAAGCAGATCCATGG
SOX2	F<br>R	TGGACAGTTACGCGCACAT<br>CGAGTAGGACATGCTGTAGGT
SOX17	F<br>R	CGCACGGAATTTGAACAGTA<br>GGATCAGGGACCTGTCACAC
EOMES	F<br>R	ATCATTACGAAACAGGGCAGGC<br>CGGGGTTGGTATTTGTGTAAGG
MIXL1	F<br>R	GGTACCCCGACATCCACTTG<br>TAATCTCCGGCCTAGCCAAA
FOXA2	F<br>R	GGGAGCGGTGAAGATGGA<br>TCATGTTGCTCACGGAGGAGTA
SOX7	F<br>R	ACGCCGAGCTCAGCAAGAT<br>TCCACGTACGGCCTCTTCTG
SOX1	F<br>R	Hs_SOX1_1_SG QuantiTect primer assay)<br>QT00215299
HAND1	F<br>R	GTGCGTCCTTTAATCCTCTTC<br>GTGAGAGCAAGCGGAAAAG
PDX1	F<br>R	AAGTCTACCAAAGCTCACGCG<br>GTAGGCGCCGCCTGC
Glucagon	F<br>R	AAGCATTTACTTTGTGGCTGGATT<br>TGATCTGGATTTCTCCTCTGTGTCT
Insulin	F<br>R	CAGGAGGCGCATCCACA<br>AAGAGGCCATCAAGCAGATCA

Supplementary Table 3: Primers used for RT-PCR to confirm pluripotency and score endodermal differentiation capacity.

hIPSC line	Neuroectoderm (NE)	Endoderm	Mesoderm
DF-iPSC-627-36-R	60-95%	Columnar endodermal epithelium	--
EP-iPSC-230-57-R	90%	5-10% endodermal epithelium<br>35% mesenchymal stroma	Few areas of cartilage
DF-iPSC-230-25-R	Dominance of NE	10% endodermal epithelium<br>20% mesenchymal stroma	5% cartilage

Supplementary Table 4: Differentiation capacity not due to culture conditions. We performed teratoma assays in 2 LDC- (EP-iPSC-230-57-R and DF-iPSC-230-25-R) and one HDC- (DF-iPSC-627-36-R) iPSC line and confirmed that LDC lines failed to make endoderm cells.

Target ID	Chr	position	primer	primer sequence	sequence to analyze
ch.16.74794652R	16	76237151	F<br>R<br>PSQ	AAATTACCTRCCATATTATTTCC<br>[Btn]TGTAAGATAAAYTTATGYTGTATTAAG<br>TRCCATATTATTTCCCTA	TARCTRCTRTAACCCA
ch.2.22266060F	2	22412555	F<br>R<br>PSQ	TTAATTGYAGAATTTGAATTTGAGT<br>[Btn]ACACACCTCATCACCTTCATCT<br>GAATTTGAATTTGAGTYTYT	YGGAGTTTAYAG
ch.1.3947147F	1	203693763	F<br>R<br>PSQ	ATAATAAGGGTYTTAGAAGTAGAAG<br>[Btn]CTCRACARCCCATARCC<br>GGTYTTAGAAGTAGAAGAGT	TTAYAAGAYAYATG
ch.19.931598F	19	24232204	F<br>R<br>PSQ	GGTYATATTTTTGATGTGTTGATT<br>[Btn]CAACRATATRTCACAATCCTTTCT<br>TTGATGTGTTGATTYTTTY	YAG
ch.16.5927259F	16	5987258	F<br>R<br>PSQ	TATGAGAGATTGTGYAGYTTTT<br>[Btn]TCTTCTCACTTCTCTCTTTCCA<br>TTYYTATAGTGTGTGGATTT	AYAGGAGYAAAGGYATYA
ch.16.785175F	16	25789762	F<br>R<br>PSQ	AGYAGGAAGGAGAAAGTTGG<br>[Btn]CACATCTTTAAAAATACCTTCACA<br>AGAAAGTTGGYTTYTYT	TAYAGYTYAAAYAAGTA
ch.17.1364760R	17	50113310	F<br>R<br>PSQ	AAATTRACAAAACTTTTCCATA<br>[Btn]TTGATGYATAGAATATTTGAGAA<br>TCATRRACTCTCARRC	TRTARTT
ch.21.579442R	21	41606280	F<br>R<br>PSQ	[Btn]TTTAACATCTCTRARCCACACA<br>AAGTATGGAAAAGYAYGTAATAG<br>GAYYATGATTTYYATTTT	AYAGGYAAAGYAA
ch.2.167882003F	2	168173757	F<br>R<br>PSQ	[Btn]TAATGTGTTTATGTYGGAGGA<br>CACATRATTCCCTTCAAA<br>RCTRATTATCTRRATCCT	RTAACATTRTCTRCAAT

Supplementary Table 5: Primer sequence and location of the nine non-CG sites examined using bisulfite pyrosequencing. Position is based on human reference assembly GRCh37.p2. F = forward primer for locus- and methylation-specific PCR, R = reverse primer for locus- and methylation-specific PCR, PSQ=locus-specific pyrosequencing primer, [Btn]=biotin label. Underlined region indicates non-CG site assayed by Illumina 450K BeadChip.

Target gene	primer	primer sequence 5´ to 3´
DNMT1	F	CCCCTGAGCCCTACCGAAT
	R	CTCGCTGGAGTGGACTTGTG
DNMT3A	F	TTCTACCGCCTCCTGCATGAT
	R	GCGAGATGTCCCTCTTGTCACTA
DNMT3B	F	GAATTACTCACGCCCCAAGGA
	R	ACCGTGAGATGTCCCTCTTGTC
DNMT3L	F	GAAGACCTGGACGTCGCATC
	R	CCTTATGGCTGGGATGTTGC
TET1	F	CAGTAAGCCTTCGTCACTGC
	R	TAAAATGGGGTTCGGTTTCA
RTL15	F	AGCCTGTCCATCATGGTGTT
	R	GATCCACTGGGTGTCAGGAT

Supplementary Table 6: Primers used for RT-PCR. DNMT1, DNMT3A and DNMT3B were taken from previously published work[1]

Supplementary Reference

1. Kurita,S., et al., DNMT1 and DNMT3b silencing sensitizes human hepatoma cells to TRAIL-mediated apoptosis via up-regulation of TRAIL-R2/DR5 and caspase-8. *Cancer Sci.* **101**, 1431-1439 (2010).
